# Optical, Structural and Paramagnetic Properties of Eu-Doped Ternary Sulfides ALnS_2_ (A = Na, K, Rb; Ln = La, Gd, Lu, Y)

**DOI:** 10.3390/ma8105348

**Published:** 2015-10-13

**Authors:** Vítězslav Jarý, Lubomír Havlák, Jan Bárta, Maksym Buryi, Eva Mihóková, Martin Rejman, Valentin Laguta, Martin Nikl

**Affiliations:** 1 Institute of Physics, Academy of Sciences of the Czech Republic, Na Slovance 1999/2, Praha 8 18221, Czech Republic; jary@fzu.cz (V.J.); havlak@fzu.cz (L.H.); buryi@fzu.cz (M.B.); mihokova@fzu.cz (E.M.); martin.rejman@centrum.cz (M.R.); laguta@fzu.cz (V.L.); 2 Faculty of Nuclear Sciences and Physical Engineering, Czech Technical University in Prague, Brehova 7, Praha 1 11519, Czech Republic; jenceslav@gmail.com

**Keywords:** luminescence, white light emitting diode, Eu^2+^, ternary sulfide, EPR

## Abstract

Eu-doped ternary sulfides of general formula ALnS_2_ (A = Na, K, Rb; Ln = La, Gd, Lu, Y) are presented as a novel interesting material family which may find usage as X-ray phosphors or solid state white light emitting diode (LED) lighting. Samples were synthesized in the form of transparent crystalline hexagonal platelets by chemical reaction under the flow of hydrogen sulfide. Their physical properties were investigated by means of X-ray diffraction, time-resolved photoluminescence spectroscopy, electron paramagnetic resonance, and X-ray excited fluorescence. Corresponding characteristics, including absorption, radioluminescence, photoluminescence excitation and emission spectra, and decay kinetics curves, were measured and evaluated in a broad temperature range (8–800 K). Calculations including quantum local crystal field potential and spin-Hamiltonian for a paramagnetic particle in D_3d_ local symmetry and phenomenological model dealing with excited state dynamics were performed to explain the experimentally observed features. Based on the results, an energy diagram of lanthanide energy levels in KLuS_2_ is proposed. Color model *xy*-coordinates are used to compare effects of dopants on the resulting spectrum. The application potential of the mentioned compounds in the field of white LED solid state lighting or X-ray phosphors is thoroughly discussed.

## 1. Introduction

Sulfide-based luminescent materials have attracted a lot of attention for a wide range of photo-, cathodo- and electroluminescent applications [1]. The lack of a bright blue phosphor to produce the third primary color was a key issue in the realization of full-color thin-film electroluminescent (FCTFE) displays until the breakthrough discovery of alkaline earth thiogallate thin films. In the 1990s, a saturated green electroluminescence was obtained with thin sputtered films of Eu^2+^-doped SrGa_2_S_4_ [2] and a deep blue one was achieved with Ce^3+^-doped SrGa_2_S_4_ and CaGa_2_S_4_ thin films [3,4]. In addition, a laser effect was observed in rare earth (RE)-doped calcium thiogallate crystals. CaGa_2_S_4_:Eu^2+^ gives rise to a 2.19 eV laser emission with unique tunable properties [5] and a mid-IR laser effect at 4.3 μm was reported for (CaGa_2_S_4_:Dy^3+^) [6]. CaGa_2_S_4_:Ce^3+^ can also be used as a gamma ray scintillator [7]. The highest light yield (*LY*) scintillating crystals are currently found among oxides ((Lu,Y)_2_SiO_5_:Ce,Ca *LY* = 32,000 ph/MeV [8], Gd_3_(Al,Ga)_5_O_12_:Ce, *LY* = 58,000 ph/MeV [9], (Gd,La)_2_Si_2_O_7_:Ce. *LY* = 41,000 ph/MeV [10]), chlorides (LaCl_3_:Ce, *LY* = 49,000 ph/MeV [11]), bromides (LaBr_3_:Ce, *LY* = 77,000 ph/MeV [12]) and iodides (SrI_2_:Eu, *LY* > 80,000 ph/MeV [13,14]. Theoretically, the maximum achievable photon yield *LY*, expressed as the number of photons emitted when 1 MeV of γ-ray energy is absorbed (ph/MeV), is proportional to the number of electron-hole pairs created by the ionizing radiation. Therefore, it is inversely proportional to the band gap of the host material. Smaller band-gap compounds such as iodides [15] and sulfides [1,16] are of interest for developing high light output scintillators. An interesting review paper describing recent research and development (R&D) trends in inorganic single-crystal scintillator materials for radiation detection was published [17]. As for the trends in the field of white light emitting diode (LED) solid state lightings, Ce^3+^ and Eu^2+^ emission centers have become of great interest recently, see for example [18,19,20,21,22].

In the presented paper, the structural, optical and paramagnetic properties of Eu-doped ternary sulfides of general formula ALnS_2_ (A = Na, K, Rb; Ln = La, Gd, Lu, Y) are investigated in great detail aiming to determine europium emission mechanisms and predict the location of lanthanide energy levels relative to the conduction and valence bands. This knowledge is helpful to predict possible loss mechanism, as it is shown, for example, for CaGa_2_S_4_ in [23]. Luminescence properties of such a material family (RE-doped ALnS_2_ sulfides) started to be studied only recently in 2011 in a pioneer work dealing with fundamental properties of RE-doped RbLaS_2_ [24], soon followed by papers on RE-doped RbGdS_2_ and RE-doped RbLuS_2_ [25,26]. It appeared that the studied materials possess a great application potential in the fields of X-ray phosphors (due to their elevated density and effective atomic number, which is, for RbLuS_2_, equal to that of Lu_3_Al_5_O_12_ (LuAG) and solid state white LED lighting (especially due to their transparency and crystal platelets nature). Surprisingly, a stable and very efficient 5d-4f Eu^2+^ emission peaking at 520 nm has been found and identified in KLuS_2_, where the lutetium cation is trivalent and the potassium cation is monovalent [27]. Charge compensation in this material has been explained by means of electron paramagnetic resonance (EPR) [28]. Ce^3+^ 5d-4f emission occurring at 580 nm in KLuS_2_ has been described in detail [29], followed by the work reviewing the optical properties of Pr^3+^, Sm^3+^, Tb^3+^ and Tm^3+^-doped KLnS_2_ (Ln = La, Gd, Lu) [30]. As a next step, doubly-doped KLuS_2_ (KLuS_2_:Eu,Ce; KLuS_2_:Eu,Pr; KLuS_2_:Eu,Sm) were presented [31], confirming the energy transfer occurrence from Eu^2+^ to the trivalent ions Ce^3+^, Pr^3+^ and Sm^3+^.

**Figure 1:**
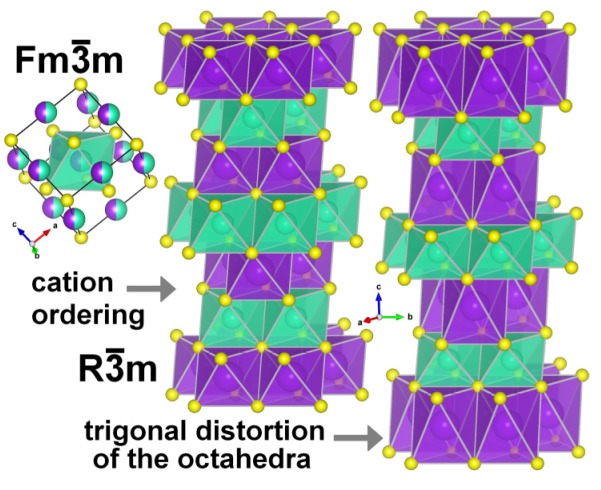
Cubic and rhombohedral modifications of ALnS_2_ sulfides. Yellow atoms: S^2−^; green atoms: Ln^3+^; pink atoms: A^+^.

Ternary sulfides with the general formula ALnS_2_ (A = Na, K, Rb; Ln = La, Gd, Lu, Y) adopt either a disordered NaCl-type cubic structure (space group Fm $\bar{3}$ m; NaLaS_2_–NaNdS_2_ (NaSmS_2_) [32,33,34]) or a layered α-NaFeO_2_-type rhombohedral structure (space group R¯3m; NaNdS_2_ (NaSmS_2_)–NaLuS_2_, KLnS_2_, RbLnS_2_ [32,33,34,35,36,37,38]). In both cubic and rhombohedral modifications, metal ions are octahedrally surrounded by six sulfur atoms in *O*_h_ or *D*_3d_ symmetry, respectively. For rhombohedral ALnS_2_ in hexagonal setting, A^+^ ions are located at Wyckoff positions 3a (0,0,0), Ln^3+^ at positions 3b (0,0,½) and sulfur ions at 6c (0,0,*z*). Both AS_6_ and LnS_6_ octahedra are trigonally distorted depending on the value of *z*—elongated or shortened, respectively; for A = Na, K, Rb and Cs, *z* is ≤¼ due to the larger size of A^+^ than Ln^3+^ (all ionic radii relevant for this work are given in Table 1). When doping the ALnS_2_ sulfides with europium, smaller Eu^3+^ should occupy the Ln^3+^ position, whereas larger Eu^2+^ can be expected either at the A^+^ site or at both A^+^ and Ln^3+^ sites. The edge-sharing octahedra are arranged into alternating layers of AS_6_ and LnS_6_ (Figure 1), which are perpendicular to the *c* axis of the crystal. Generally, the α-NaFeO_2_-type ALnS_2_ sulfides form hexagonal platelets with the *c* axis perpendicular to their flat sides [16]. The structure of several ALnS_2_ ternary sulfides was recently determined or re-determined [35,37,38] due to their potential application as luminescent materials and dubious values of *z* reported in the literature.

The size and shape of coordination polyhedron has a large effect on emission properties of 5d-4f emitting ions such as Ce^3+^ or Eu^2+^. All structural parameters of the discussed sulfides that may be important for Eu^2+^ 5d-4f emission are summarized in Table 2, including bond lengths *d*(X–S), thickness of the respective layer *t*(XS_6_) and angles φ_1,2_(X) between S–X–S, where X stands for either A or Ln. As can be seen from the table, the value of *a* mainly reflects the lanthanide ion, whereas *c* is more influenced by the alkali metal ion.

In this work we present a novel material family, Eu^2+^-doped ALnS_2_, which represents a new material concept for solid state white LED lighting based on suitably positioned Eu^2+^ absorption bands in the near UV and blue spectral region, very intense emission peaking from 495 nm (RbLuS_2_:Eu) to 779 nm (NaGdS_2_:Eu), fast room temperature decay time (~400–700 ns) and very good thermal stability up to 200 °C. Structural, optical and paramagnetic properties of Eu^2+^ activator in these hosts are investigated in great detail and it is the aim of the presented work to explain and clarify experimentally obtained data by using proper physical models.

**Table 1:** Octahedral ionic radii of the relevant ions.

Ion	Ionic Radius * (Å)	Ion	Ionic Radius * (Å)
La^3+^	1.032	Na^+^	1.02
Gd^3+^	0.938	K^+^	1.38
Y^3+^	0.900	Rb^+^	1.52
Lu^3+^	0.861	Eu^2+^	1.17
Eu^3+^	0.947	S^2−^	1.84

* for coordination number six, after [39].

**Table 2:** Structural parameters of all discussed ALnS_2_ sulfides.

Compound	*a* (Å)	*c* (Å)	*z*	*d^2^*(A–S) (Å) *	*d^2^*(Ln–S) (Å) *	*t*(AS_6_) (Å) **	*t*(LnS_6_) (Å) **	φ_1_(A) ***	φ_1_(Ln) ***
NaLaS_2_ [37]	5.877	-		2.938		3.393		90°	
NaGdS_2_ [38]	4.014	19.878	0.2433	2.928	2.773	3.579	3.047	86.5°	92.7°
NaYS_2_ [38]	3.96	19.867	0.2426	2.912	2.739	3.605	3.017	85.7°	92.6°
NaLuS_2_ [38]	3.891	19.85	0.2415	2.893	2.693	3.647	2.969	84.5°	92.5°
KLaS_2_ [37]	4.265	21.929	0.2372	3.242	2.908	4.217	3.093	82.3°	94.3°
KGdS_2_ [37]	4.072	21.901	0.235	3.188	2.787	4.307	2.994	79.4°	93.9°
KYS_2_ [37]	4.022	21.884	0.2344	3.174	2.755	4.328	2.966	78.6°	93.7°
KLuS_2_ [37]	3.949	21.871	0.2337	3.154	2.711	4.359	2.932	77.5°	93.5°
RbLaS_2_ [36]	4.296	22.93	0.2337	3.372	2.918	4.569	3.074	79.1°	94.8°
RbGdS_2_ [36]	4.11	22.9	0.232	3.319	2.805	4.641	2.992	76.5°	94.2°
RbYS_2_ [37]	4.044	22.827	0.2309	3.304	2.757	4.676	2.932	75.5°	94.3°
RbLuS_2_ [36]	3.991	22.838	0.2303	3.293	2.724	4.706	2.907	74.6°	94.2°

* *d*^2^(X–S) = ⅓∙*a*^2^ + ¼∙*t*^2^(XS_6_); ** *t*(AS_6_) = 2∙*c*∙(⅓ − *z*), *t*(LnS_6_) = *c*∙(2∙*z* − ⅓); *** φ_1_ = 180° − φ_2_ = cos^−1^ {[*d*^2^(X–S) − ½∙*a*^2^]/*d*^2^(X–S)}.

## 2. Experimental Section

### 2.1. Sample Preparation

Starting raw materials were carbonates: Na_2_CO_3_ (Alfa Aesar, ≥99.95%, Karlsruhe, Germany), K_2_CO_3_ (Alfa Aesar, ≥99.997%), Rb_2_CO_3_ (Alfa Aesar, ≥99.8%) and oxides: La_2_O_3_ (Koch-Light Laboratories, ≥99.999%, Colnbrook, UK), Gd_2_O_3_ (Koch-Light Laboratories, ≥99.999%), Lu_2_O_3_ (Fluka, ≥99.999%, Buchs, Switzerland), Y_2_O_3_ (Fluka, ≥99.999%), Eu_2_O_3_(Alfa Aesar, ≥99.99%). Used gases were Ar (Linde, ≥99.999%, Prague, Czech Republic) and H_2_S (Linde, ≥99.5%, Pullach, Germany). Starting materials for the Eu-doped compounds were mixtures of alkali metals carbonates (A_2_CO_3_) and rare-earth oxides doped by europium in the molar ratio 80:1. Rare-earth oxides were doped by europium by mixing and thorough homogenization of the Ln_2_O_3_ (Ln = La, Gd, Lu and Y) and Eu_2_O_3_ mixture.

The chemical reactions were realized either in the corundum (Haldenwanger, ≥99.7% Al_2_O_3_, Waldkraiburg, Germany) or sapphire single-crystalline tube (Crytur, ≥99.99% Al_2_O_3_, Turnov, Czech Republic). The sapphire single-crystalline tube (Crytur) appeared to be more resistant and suitable for higher temperatures. The tube was put into an electric resistance furnace equipped with the heating/cooling speed rate regulation. The scheme of the setup is outlined in [24]. Either Ar or H_2_S gases are then introduced into the reaction tube volume. They are taken directly from the pressurized bottles using a three-way cock to switch between them. Prior to the reaction itself, starting material mixtures (A_2_CO_3_ and Ln_2_O_3_:Eu) were mixed and homogenized in agate mortar. The prepared mixture was placed in a corundum boat and put into the corundum (or sapphire) tube (inner volume of which is around 0.9 dm^3^). The reaction mixture was then heated up to 1000 °C for potassium and rubidium compounds and up to 1200 °C for sodium compounds using an electric resistance furnace (heating rate 10 °C/min) under the flow of argon gas (15 dm^3^/h). When the desired temperature was reached, the reaction mixture was annealed for 60–120 min under the flow of hydrogen sulfide (15 dm^3^/h). Straight after annealing, the reaction system was cooled under the flow of Ar (1 °C/min, 0.3 dm^3^/h). Upon reaching room temperature (RT), the corundum boat was removed from the tube furnace and the reaction products were treated by a decantation process (three times by distilled water and once by alcohol). Thus, binary alkali metal sulfides dissolved in the water. The weighing of the final product showed that the reaction conversion reached almost 100% and never dropped below 95%. The losses were caused by imperfect product separation. The product was stored in small glass flasks under an Ar atmosphere and used for further analysis.

Ternary sulfide ALnS_2_ is created at the given temperatures (see below) according to Equation (1) while the excess of A_2_CO_3_ reacts as Equation (2):

(1) A_2_CO_3_ (l) + Ln_2_O_3_ (s) + 4 H_2_S (g) → 2 ALnS_2_ (s) + 4 H_2_O (g) + CO_2_ (g)

(2) A_2_CO_3_ (l) + H_2_S (g) → A_2_S (l) + H_2_O (g) + CO_2_ (g)

Based on the melting points of binary alkali metal sulfides, it is possible to estimate the reaction temperatures needed for ALnS_2_ production. If the reaction temperature is lower than the melting point of the binary sulfide, the surface of melted carbonates solidifies during the reaction with H_2_S. The melting points of alkali metal sulfides are 1168 °C for Na_2_S [40], 948 °C for K_2_S [41], 750 ± 200 °C for Rb_2_S [42]. The phase diagrams of A–S systems can also be found in a respective work [40,41,42]. For the KLnS_2_ and RbLnS_2_ preparation, the minimal reaction temperature is around 1000 °C. Reaction time increases from La to Lu, which is probably due to the increasing melting points of Ln oxides, from La_2_O_3_ to Lu_2_O_3_. Minimal reaction time for the starting materials mixture of 10 g with given H_2_S flow is from 1 to 2 h. For the NaLnS_2_ preparation, the required reaction temperature is around 1200 °C. Under such circumstances, the sintered corundum tube (Haldenwanger) would be severely damaged and therefore the reaction must be carried out in the single-crystalline sapphire tube (Crytur). At lower temperatures, the product contains a mixture of Ln_2_O_2_S, NaLnS_2_ and Ln_2_O_3_.

### 2.2. Experimental Setup

The phase composition of thoroughly ground samples was determined by X-ray powder diffraction using the Rigaku MiniFlex 600 diffractometer (Cu anode, NaI(Tl) detector, glass sample holders with 0.2 mm depression; Rigaku Corporation, Tokyo, Japan) and ICDD PDF-2 structural database (International Centre for Diffraction Data, Powder Diffraction File, version 2013). The X-ray fluorescence analyzer Niton XL3t 900 Series (Thermo Fisher Scientific, Waltham, MA, USA) with geometrically optimized large area drift detector (GOLDD) technology was employed to investigate the elemental composition of samples and identify low-concentration impurities.

Absorption spectra were measured using the ultraviolet/visible/near infrared (UV/VIS/NIR) Spectrophotometer Shimadzu 3101PC. Radioluminescence (RL), photoluminescence excitation (PLE) and emission (PL) spectra and decay curves were measured by a custom-made spectrofluorometer 5000M (Horiba Jobin Yvon, Wildwood, MA, USA), using a steady state deuterium lamp (PL and PLE spectra), Mo X-ray tube (RL spectra), microsecond xenon pulsed flash lamp (slow or delayed recombination decays) or nanosecond nanoLED pulsed light sources (fast prompt decay curves) as the excitation sources. The detection part of the setup involved a single-grating monochromator and a photon counting detector TBX-04. Measured spectra were corrected for the spectral dependence of excitation energy (PLE) and spectral dependence of detection sensitivity (PL). Convolution procedure was applied to the decay curves to determine true decay times (SpectraSolve software package, Ames Photonics). Measurements of the optical characteristics within the 8–800 K temperature regions were performed using a closed cycle refrigerator (Janis instruments, Wildwood, MA, USA).

Continuous wave (CW) EPR measurements were performed by a Bruker X-/Q-band E580 FT/CW ELEXSYS spectrometer (Bruker Corporation, Billerica, MA, USA) at X,Q-bands with the microwave frequencies 10 and 34 GHz, respectively, in the temperature range 10–298 K. Angular variations of the spectra were carried out with a step of 2.5°–5° by using a standard goniometer.

In the following part of the manuscript, expression ALnS_2_:Eu will be used to denote ALnS_2_:Eu (A = Na, K, Rb; Ln = La, Gd, Lu, Y; 0.05% Eu dopation).

## 3. Results and Discussion

In the following part of the paper structural (Section 3.1), optical (Section 3.2) and paramagnetic (Section 3.4) properties of ALnS_2_:Eu are described in great detail with the aim to understand their mutual relations. Occurrence of low temperature Eu^3+^ emission is discussed in Section 3.3. Furthermore, the obtained data are used to construct an energy level diagram (Section 3.5). Finally, CIE coordinates (Commission Internationale de I’Eclairage) are presented in Section 3.6.

### 3.1. Structural Properties

**Figure 2:**
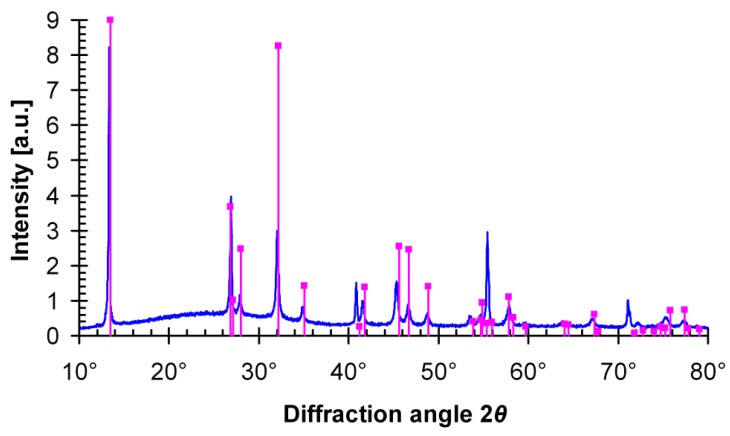
Diffraction pattern of prepared NaLuS_2_:Eu sample compared with ICDD PDF-2 record.

According to the measured diffraction patterns of the powdered ALnS_2_:Eu samples (e.g., NaLuS_2_:Eu—see Figure 2) and single-crystal X-ray diffraction measurements on undoped crystals [37,38], the formed hexagonal platelets consist only of α-NaFeO_2_-type rhombohedral ALnS_2_ (except for cubic NaLaS_2_, where the NaCl-type cubic lattice was observed). The diffraction line positions corresponded well to the expected values of *a* and *c* reported in the literature (Table 2). Despite thorough grinding of crystals, strong preferential orientation of crystals was observed (increased intensity of (0 0 n) lines in Figure 2, where n is an integer) because the thin and flat platelets easily orient themselves parallel to any flat surface. For luminescence and EPR measurements, the largest available crystals were always selected to reduce the effect of light scattering.

### 3.2. Fundamental Optical Properties

**Figure 3:**
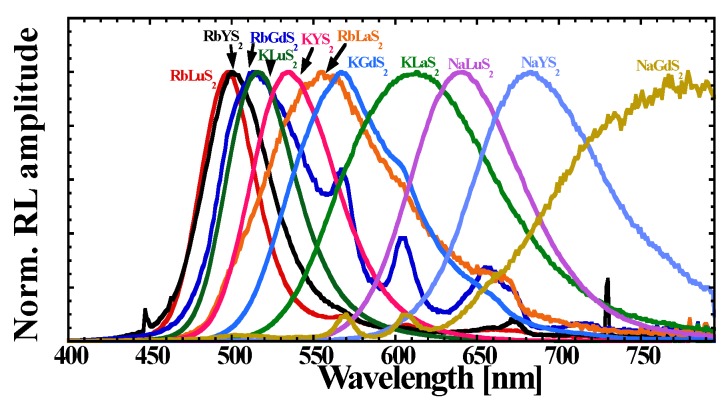
Room temperature radioluminescence (RT RL) spectra (40 kV, 15 mA) of ALnS_2_:Eu; data of KLuS_2_:Eu after [27].

Room temperature (RT) RL spectra of ALnS_2_:Eu are shown in Figure 3. All the spectra are dominated by a broad band, which we assign to the dipole allowed Eu^2+^ 5d-4f transition, also based on our previous work [27]. The positions of the maximum shifts from 498 nm (RbLuS_2_:Eu) to 779 nm (NaGdS_2_:Eu), for details see Table 3, are most probably due to the changes in the crystal field strength of different sulfide hosts (see below). It is to be noted that the europium emission in RbLaS_2_:Eu (1%) was claimed to be quenched at RT [24], probably due to heavy concentration quenching as ntense Eu^2+^ emission is observed here (0.05% sample). This is fully supported by the concentration dependence measurement performed [27]. There is a trend of the RT RL intensity reduction in the series ALuS_2_-AYS_2_-AGdS_2_-ALaS_2_ (in the sense of increasing Ln^3+^ radius [39]) for all the A = Rb, K, Na cations. A comparison in the Rb-K-Na series only at RT is rather speculative as a different degree of thermal quenching and/or ionization can occur. The RL spectrum of RbGdS_2_:Eu is partially contaminated by the Sm^3+^ 4f-4f emission lines in the 550–750 nm region. Scintillation light yield of 35.000 ph/MeV for KLuS_2_:Eu (0.05%) has been shown [16], which, together with high RL intensity compared to Bi_4_Ge_3_O_12_ (BGO) standard, allows the usage of the Eu^2+^-doped ALnS_2_ compounds as X-ray/γ-ray phosphors.

**Figure 4:**
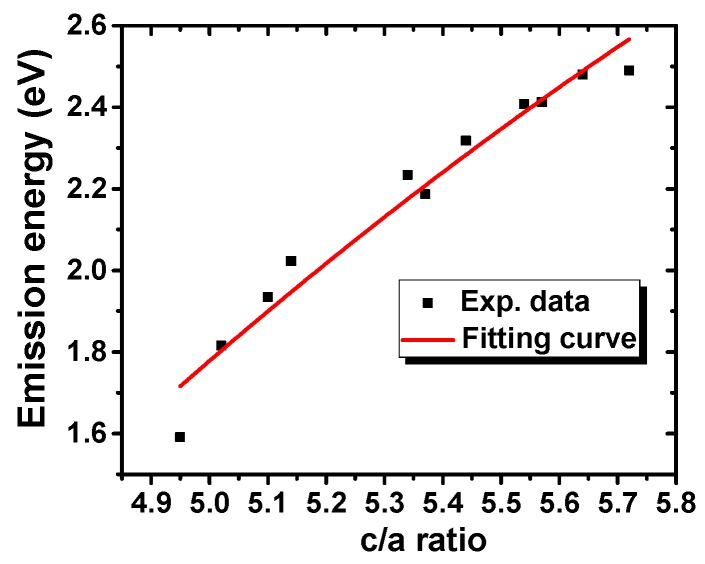
Emission maxima as a function of hexagonality (*c*/*a*) for the ALnS_2_:Eu.

NaLaS_2_:Eu sample shows no RT Eu^2+^ emission under the X-ray excitation which may be caused by its crystallization in a cubic structure instead of the rhombohedral structure. Another possible explanation takes into account the fact that the emission can be positioned even beyond 800 nm, where our instrumental setup is insensitive. However, for ALnS_2_:Eu samples crystallizing in the rhombohedral structure, rather interesting dependence of emission wavelength on their hexagonality (*c*/*a*) was found for the first time, see Figure 4. The observed positions of Eu^2+^ 5d-4f emission band(s) in ALnS_2_ should be related to their crystalline structure and crystal field. Thus, correlation between the energy of Eu^2+^ emission peak maximum *E*_em_ and structural parameters (Table 2) was sought. In the plot of *E*_em_ *versus* either *d*(Ln–S) or *d*(M–S), large discontinuities occur between ALnS_2_ with different A, so the Eu^2+^ 5d-4f emission energy cannot be a simple function of *d*. However, the *c*/*a* ratio (hexagonality, Figure 4) and the S–A–S angle around the alkali metal ion φ_1,2_(A) were found to be strongly correlated to *E*_em_. The dependence on *c*/*a* was investigated according to crystal field theory.

**Table 3:** Optical properties of ALnS_2_:Eu.

Compound	Eu^2+^ 5d-4f Emission Maximum (nm (eV))	*c*/*a* Ratio	% BGO at RT	Band Gap at RT (nm (eV))	RT PL Decay Time (ns)	Eu^2+^ 4f-5d Excitation Maximum (nm (eV))
RbLuS_2_	498 (2.49)	5.72	102	310 (4.00)	553	389 (3.19)
RbYS_2_	500 (2.48)	5.64	72	307 (4.04)	514	393 (3.16)
RbGdS_2_	514 (2.41)	5.57	26	321 (3.86)	453	391 (3.17)
RbLaS_2_	555 (2.23)	5.34	18	323 (3.84)	513	390 (3.18)
KLuS_2_ [27]	515 (2.41)	5.54	1765	308 (4.03)	454	396 (3.13)
KYS_2_	535 (2.32)	5.44	614	309 (4.01)	496	393 (3.16)
KGdS_2_	567 (2.19)	5.38	531	330 (3.76)	437	394 (3.15)
KLaS_2_	613 (2.02)	5.14	126	325 (3.82)	689	394 (3.15)
NaLuS_2_	641 (1.93)	5.10	774	304 (4.08)	488	429 (2.89)
NaYS_2_	683 (1.82)	5.02	119	309 (4.01)	511	437 (2.84)
NaGdS_2_	779 (1.59)	4.95	25	330 (3.76)	531	~430 (2.88)

Experimental data of emission energy as a function of *c*/*a* hexagonality (the values of which are listed in Table 3) were fitted by Equation (3) in the form:

(3) Eem=Δ−A′(1+(ξca)2)3/2−B′(1+(ξca)2)5/2

where ξ^2^ = 1/48 ≈ 0.0208; Δ, $A^{\prime }$ and $B^{\prime }$ are fitting parameters. Their meanings as well as the derivation of Equation (3) are discussed in Supplementary Materials. From the fit the following values were obtained: Δ = 4.7 ± 0.2 eV, $A^{\prime }=-4.4\pm 1$, $B^{\prime }=-14.4\pm 2.$

**Figure 5:**
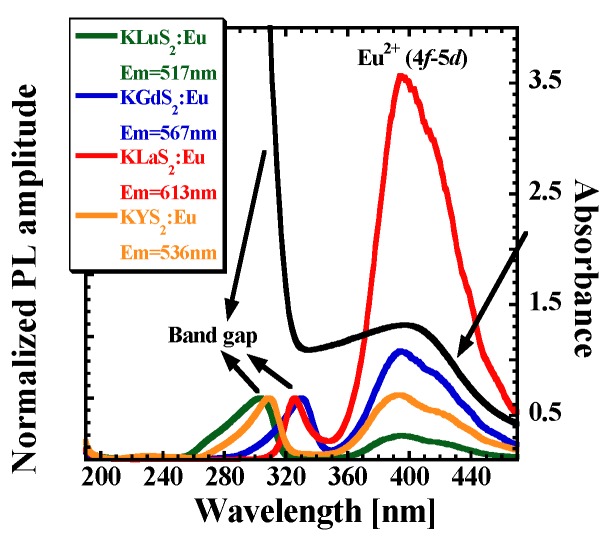
RT PLE spectra of KLnS_2_:Eu (0.05%) samples (Ln = Lu, Y, Gd, La) and RT absorption spectra of KLuS_2_:Eu (2% Eu, thickness 0.2 mm); data of KLuS_2_:Eu after [27].

As an example, RT PLE spectra of Eu^2+^-doped KLnS_2_:Eu (0.05%; Ln = Lu, Y, Gd, La) are presented in Figure 5. The emission wavelengths used for the PLE spectra recording were taken from the RL spectra maxima, see Figure 3 and Table 3. All PLE spectra feature the KLnS_2_ band edge shifting between 308 nm (KLuS_2_) and 330 nm (KGdS_2_), which is in a fairly-good agreement with previously reported values [27,30], and another band at lower energies ascribed to the Eu^2+^ 4f-5d transition, similarly to [27]. Such a band is present in all studied samples ALnS_2_:Eu (not shown here). Its position covers the range from 389 nm (RbLuS_2_:Eu) to 437 nm (NaYS_2_:Eu). Interestingly, for the RbLnS_2_ and KLnS_2_ compounds, only a very small variation in the band position is observed (389–396 nm) while for the NaLnS_2_ series, low energy shift is observed (429–437 nm). The corresponding transition is partially allowed and represents an interesting way of efficient excitation in the near UV/blue region. Obviously, absorption spectra would provide better understanding, but since we are dealing with low Eu concentration (to avoid any concentration quenching effects) and the NaLnS_2_:Eu (Ln = Lu, Y, Gd) crystals are very small, it is unfeasible to measure well-resolved absorption spectra. However, an example of the absorption spectrum of KLuS_2_:Eu (2%) is displayed in Figure 5, showing good correlation between absorption and excitation features.

RT decay curves related to the Eu^2+^ 5d-4f transitions in ALnS_2_:Eu (λ_ex_ and λ_em_ taken from the maxima of RL and PLE spectra, see Figure 3 and Figure 5, Table 3) can be fitted by a single exponential to the initial decrease. The decay time values are listed in Table 3. All values are in the order of a few hundred nanoseconds which is in a good agreement with the expected value of dipole allowed 5d-4f Eu^2+^ transitions.

As an example, four normalized decay curves of KLnS_2_:Eu (Ln = Lu, Y, Gd, La; 0.05% Eu) are shown in Figure S13 in the Supplementary File (Luminescence and EPR experiment—additional data). Interestingly, their signal-to-background ratio improves in the KLuS_2_:Eu-KYS_2_:Eu-KGdS_2_:Eu-KLaS_2_:Eu series, which may be related to processes of the excited state ionization of the Eu^2+^ activator, at least in the KGdS_2_, KLaS_2_ hosts, see below.

**Figure 6:**
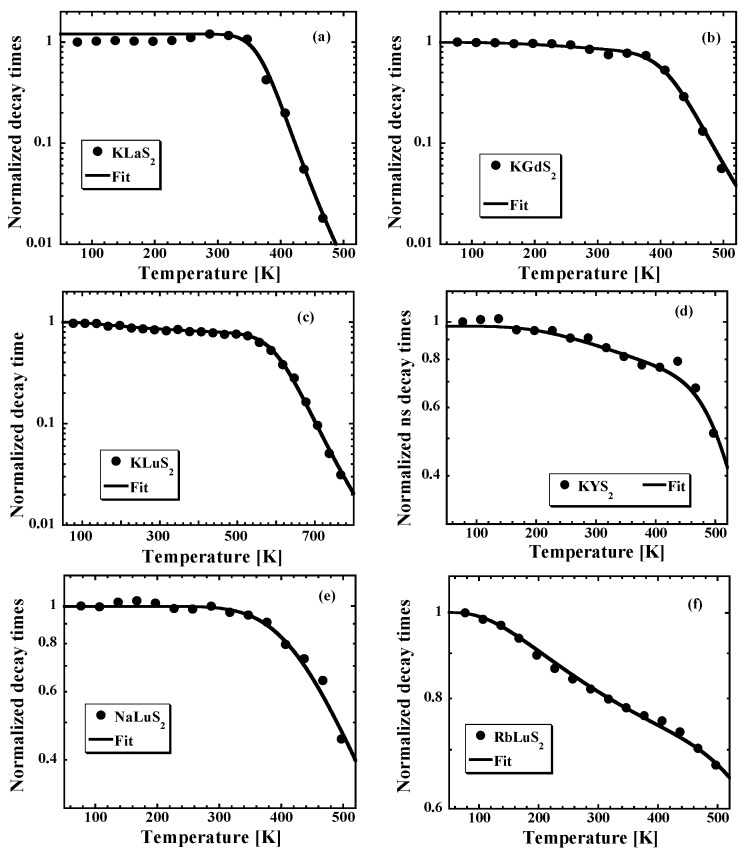
Temperature dependence of the emission (PL) decay times of Eu^2+^ in (**a**) KLaS_2_; (**b**) KGdS_2_; (**c**) KLuS_2_ after [16,27]; (**d**) KYS_2_; (**e**) NaLuS_2_ and (**f**) RbLuS_2_ hosts; solid symbols are experimental data, solid lines are fits to the data using the phenomenological model described in the text. The parameters of fits are summarized in Table 4.

To further study the thermal stability of the Eu^2+^ emission center in these ternary sulfide hosts, the temperature dependences (TDs) of the Eu^2+^ 5d-4f decay times in KLnS_2_ hosts (Ln = Lu, Gd, Y, La) and ALuS_2_ hosts (A = Na, K, Rb) were investigated between 77 and 800 K (see Figure 6). Radiative lifetime values (at 77 K, not effected by any quenching or ionization processes) are listed in Table 4, together with the excitation and emission wavelengths. Lu-compounds appear to be the most thermally stable as the decay time values at 497 K still reach 80%, 70% and 45% of their low-temperature limit for KLuS_2_ (already reported [27]), RbLuS_2_ and NaLuS_2_, respectively. Furthermore, prolonged TD of Eu^2+^ decay curves in KLuS_2_ up to 770 K shows that the decay time value even at 770 K is 18 ns [16]. On the other hand, thermal stability decreases in the KLuS_2_-KYS_2_-KGdS_2_-KLaS_2_ series as the decay time values decrease by more than two orders of magnitude between 77 and 497 K in KLaS_2_:Eu.

We approximated the mentioned nanosecond decay time TDs by a simple barrier model described by:

(4) $$\frac{1}{{\text{ô}}_{\text{observed}}}=\frac{1}{{\text{ô}}_{\text{radiative}}}+\sum^{\text{​}}K_{x_{i}}e^{-\frac{E_{x_{i}}}{kT}}$$

where ô_observed_, ô_radiative_, *K_xi_*, *E_xi_*, *k* and *T* represent the PL decay time measured at temperature *T*, the low-temperature limit of the PL decay time (see Table 4), frequency factor of the *i*-th escaping channel, *i*-th energy barrier height, Boltzmann constant and absolute temperature, respectively. The parameters of the best fit of Equation (4) to the experimental data are listed in Table 4. As already published [27], the low value of the energy barrier (40 meV in KLuS_2_:Eu) indicates that the decay time shortening in KLuS_2_:Eu (up to 497 K) is not due to a classical temperature quenching to the ground state. It can be caused by a transition to some other state, perhaps that of a nearby defect. In RbLuS_2_:Eu, this escaping channel with the *ca.* 40 meV energy barrier reported in KLuS_2_:Eu can be found as well, but also another one with the energy barrier of 500 meV appears. This channel we ascribe to classical thermal quenching and/or thermally induced ionization of the Eu^2+^ 5d excited state (in the 77–497 K temperature range). Such a process starts to play a role in KLuS_2_:Eu as well at temperatures above 500 K and the corresponding energy barrier is 820 meV. On the other hand, NaLuS_2_:Eu can be reasonably fit with a single escaping channel with the energy barrier 300 meV (see Table 4). TD of the Eu^2+^ decay times in KYS_2_:Eu and KGdS_2_:Eu exhibits a similar behavior as KLuS_2_:Eu and again can be fit with a model introducing two escaping channels (described above). Finally, TD of the Eu^2+^ nanoseconds (ns) decay time in KLaS_2_:Eu can be approximated by a single barrier model with the energy value of 650 meV and very high frequency factor (9 × 10^14^ s^−1^—see Table 4).

**Table 4:** Emission (PL) decay time temperature dependences and fit parameters of Eu^2+^ in a selection of ALnS_2_:Eu. λ_exc_, λ_em_, ô_rad_, K*_ix_* and E*_ix_* are excitation and emission wavelengths, low-temperature limit of observed radiative lifetime, frequency factors and energy barriers of the emission quenching channels. For more details, see the text.

Host	λ_exc_ (nm)	λ_em_ (nm)	ô_rad_ (ns)	*K*_1*x*_ (s^−1^)	*E*_1*x*_ (meV)	*K*_2*x*_ (s^−1^)	*E*_2*x*_ (meV)
KLaS_2_	389	610	573	-	-	9 × 10^14^	650
KYS_2_	389	536	546	5 × 10^6^	80	1 × 10^13^	700
KGdS_2_	389	550	517	3 × 10^6^	60	2 × 10^13^	580
KLuS_2_ [16,27]	389	517	526	1.4 × 10^6^	40	1.2 × 10^13^	820
RbLuS_2_	389	500	675	1.6 × 10^6^	40	1 × 10^10^	500
NaLuS_2_	452	635	489	-	-	2.5 × 10^9^	300

To further investigate the nature of decay times shortening at higher temperatures, the measurement of the TD of the delayed recombination (DR) integrals was performed. This measurement consists in monitoring of the decay under direct optical excitation of the emission center using a xenon-filled flash-lamp in multichannel scaling mode while collecting the emission light in an extended time window (88 ms). Under such conditions, prompt nanosecond Eu^2+^ decay does not have to be taken into account and only the delayed light (produced by electrons that were thermally ionized into the conduction band, and later returned back to the emission center) can be easily investigated (for details concerning the method see [43]).

**Figure 7:**
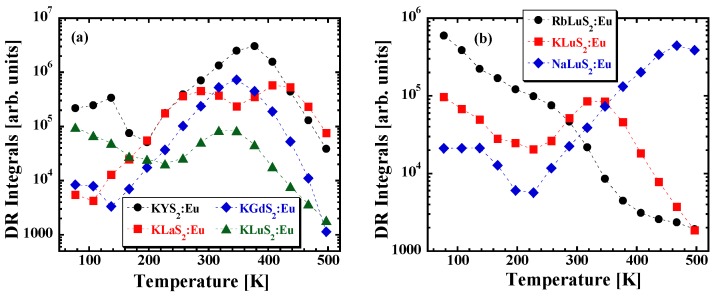
Temperature dependence (TD) of the delayed recombination (DR) integrals (excitation and emission wavelengths identical to those for nanoseconds decay time measurements, see Table 4) for (**a**) KLnS_2_:Eu (Ln = La, Gd, Lu, Y) and (**b**) ALuS_2_:Eu (A = Rb, K, Na); composition given in the legend.

**Figure 8:**
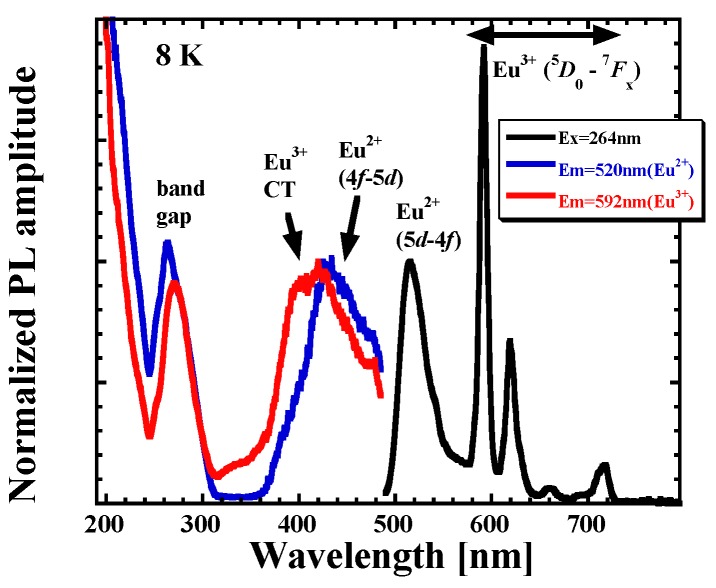
PL and photoluminescence excitation (PLE) spectra of KLuS_2_:Eu^2+^ (0.05%) recorded at 8 K.

Figure 7 illustrates the TD of the DR integrals related to the Eu^2+^ center in different sulfide hosts. Before integrating the decay curves, a few points with the highest intensity at the very beginning of the decay (containing prompt Eu^2+^ ns luminescence) were omitted, for details see [44]. As demonstrated in Figure 8 there is, indeed, an increase of the DR integrals between 200–380 K, 140–340 K, 100–440 K, 200–300 K, 200–480 K for KYS_2_:Eu, KGdS_2_:Eu, KLaS_2_:Eu, KLuS_2_:Eu^27^ and NaLuS_2_:Eu, respectively. We tentatively ascribe it to a process in which the electron escapes from the Eu^2+^ 5d excited state to either a nearby defect or to a conduction band, from where it can return at later times and radiatively recombine with the hole, giving rise to the DR luminescence. The hypothesis of the nearby defect being involved is supported by the low value of the energy barrier found above, especially for the KLuS_2_:Eu, KGdS_2_:Eu, RbLuS_2_:Eu and KYS_2_:Eu. Rapid decrease of the DR integrals at higher temperatures can be due to the shaping of the DR temperature dependence by the presence of traps [45,46]. An exception from the behavior is to be noted for the RbLuS_2_:Eu, as there is a decrease of the DR integrals in the whole temperature range (77–497 K). We also note that DR integrals show a non-zero value even at the lowest temperatures, which has been explained by quantum tunneling between the luminescence center and a nearby defect state [47]. Better understanding of the DR behavior, however, would require an independent study of characteristics of the traps involved in the DR process as mentioned above.

### 3.3. Eu^3+^ Emission at Low Temperatures

**Figure 9:**
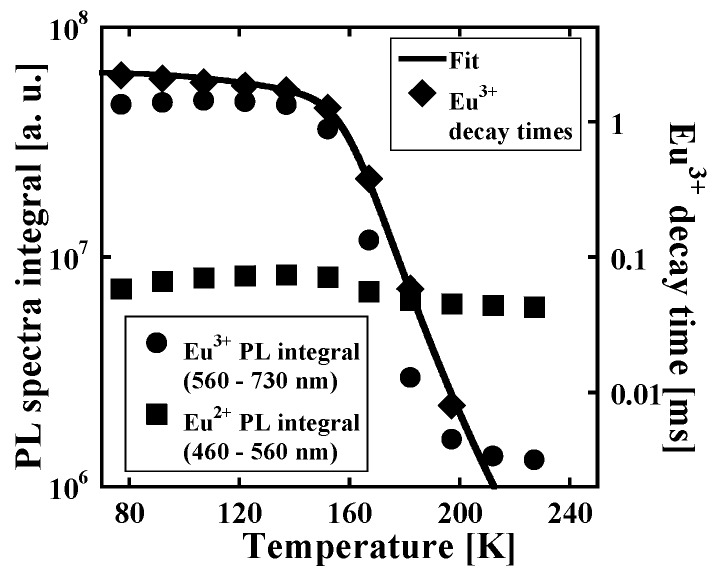
Temperature dependence of PL spectra integrals, separately for Eu^3+^ (560–730 nm) and Eu^2+^ (460–560 nm) emission region (see Figure 9) under 390 nm excitation and Eu^3+^ decay times (λ_ex_ = 390 nm, λ_em_ = 592 nm) with fit by the phenomenological model (see Equation (4)) of KLuS_2_:Eu (0.05%).

To our surprise, PL spectrum of KLuS_2_:Eu (0.05%) recorded at 8 K uncovered the presence of the characteristic ^5^*D*_0_-^7^*F*_x_ emission lines in the 570–730 nm spectral region assigned to the Eu^3+^, see Figure 8, co-existing with the known 5d-4f Eu^2+^ emission at 515–520 nm. Mentioned Eu^3+^ emission starts to vanish above 150 K as demonstrated in Figure 9, where the temperature dependence of Eu^3+^ emission spectra integrals (PL spectra under the 390 nm excitation integrated in the 560–730 nm region) is displayed (full circles). At around 200 K, the Eu^3+^ emission is no longer observed and the Eu^2+^ emission band dominates the spectrum completely. At the same time, the Eu^3+^ decay time at the lowest temperature reaches a value of around 2.5 ms, which is typical for the parity forbidden 4f-4f RE^3+^ transitions. However, the decay times start to decrease drastically above 150 K and at around 200 K the decays become undetectable (ô at 197 K is ~8 μs), which is well in agreement with PL integral behavior. PL integrals of Eu^2+^ under the 390 nm excitation, integrated in the 460–560 nm region, however, remain constant in the studied temperature range, which implies that Eu^2+^ and Eu^3+^ centers are probably independent, as decreasing Eu^3+^ emission does not enhance the Eu^2+^ emission. TD of Eu^3+^ decay times was also fit by the phenomenological model described above, yielding the values of parameters *K*_1*x*_ = 1 × 10^4^, *E*_1*x*_ = 50 meV, *K*_2*x*_ = 3 × 10^14^, *E*_2*x*_ = 370 meV. Eu^3+^ heavy quenching in the 150–200 K region is therefore governed by the process with energy barrier of 370 meV height. The nature of the described observation is discussed in Section 3.5.

Similar behavior was also observed for KYS_2_:Eu, KGdS_2_:Eu, NaLuS_2_:Eu, RbLuS_2_:Eu (all 0.05% concentration) even for the band-gap and X-ray excitation. Interestingly, the Eu^3+^ emission is fully absent even at the lowest temperatures (8 K) in KLaS_2_:Eu. To further investigate both divalent and trivalent europium behavior, low-temperature (8 K) PLE spectra were measured separately for Eu^3+^ (λ_ex_ = 592 nm) and Eu^2+^ emission (λ_em_ = 520 nm) in KLuS_2_. Both spectra feature the band-gap related maximum below 300 nm. While the latter spectrum shows the already known 4f-5d Eu^2+^ band positioned at 430 nm (which is low-energy shifted with respect to room temperature), the former features a new band at around 400 nm, which we ascribe to a charge transfer (CT) transition of Eu^3+^ (S^2−^-Eu^3+^), based also on [48]. This assignment is discussed in Section 3.5 (energy diagram).

### 3.4. EPR Study

For the detailed EPR study, only the KLnS_2_:Eu (Ln = Lu, La, Y) ternary sulfides were chosen, since they reveal strong enough signals from the Eu^2+^ paramagnetic centers. In the NaLuS_2_:Eu, even at the Q band (34 GHz) only the central +1/2 ↔ −1/2 spin transition appears in the spectra, which does not allow any valuable information about the structure of the Eu^2+^ centers as compared to the KLuS_2_:Eu [28]. In the sulfides of the general formula AGdS_2_:Eu (A = Na, K or Rb), the signals from the Eu^2+^ ions cannot be detected separately, as the Eu^2+^ ions are coupled with the Gd^3+^ lattice ions by exchange and magnetic dipole interaction. As a result, only a very broad signal from the coupled ions is detected.

**Figure 10:**
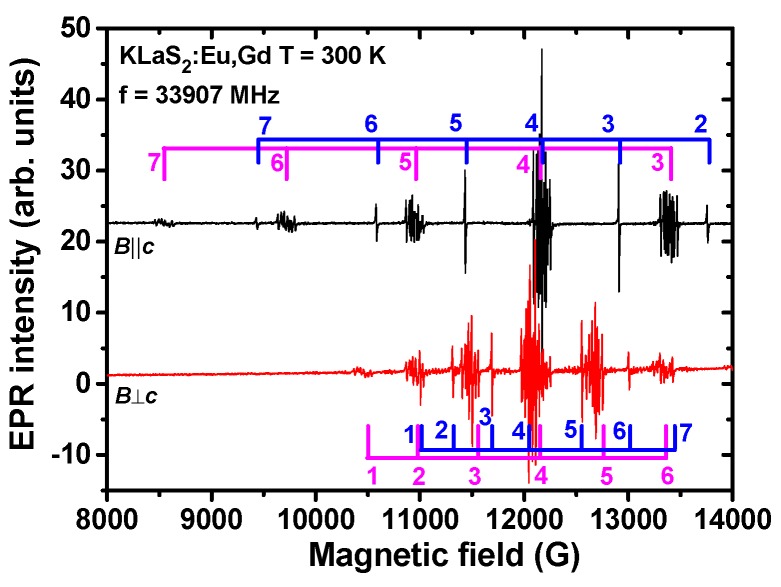
Electron paramagnetic resonance (EPR) spectra measured in KLaS_2_:Eu single crystal at two magnetic field directions, ***B***||***c*** and ***B***⊥***c***. The pink combs indicate transitions corresponding to Eu^2+^ (each transition is characterized by a pronounced hyperfine structure (HFS)) and the blue combs indicate transitions corresponding to Gd^3+^ (single narrow lines). Numbers are assigned to particular transitions; 1: −7/2 ↔ −5/2, 2: −5/2 ↔ −3/2, 3: −3/2 ↔ −1/2, 4: −1/2 ↔ +1/2, 5: +1/2 ↔ +3/2, 6: +3/2 ↔ +5/2, 7: +5/2 ↔ +7/2.

**Figure 11:**
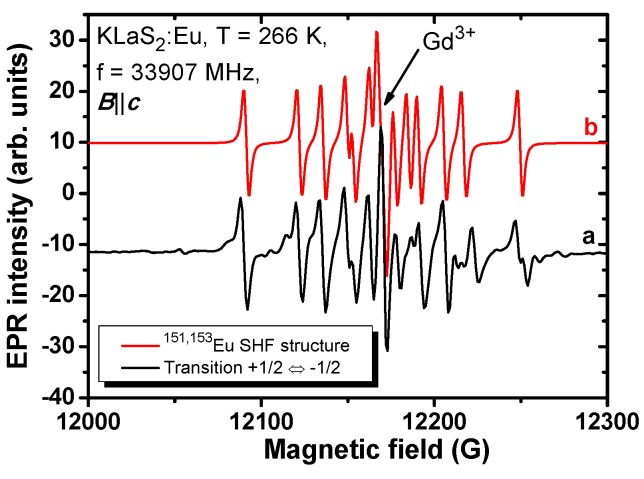
Experimental (**a**) and simulated (**b**) EPR spectra of the Eu^2+^ centra transition +1/2 ↔ −1/2 showing hyperfine structure from ^151,153^Eu isotopes.

EPR spectra measured in the Eu-doped KLaS_2_ and KYS_2_ show resonance lines produced by not only Eu^2+^ but Gd^3+^ ions (uncontrolled impurity) as well (see, e.g., Figure 10). Each of the Eu^2+^ fine components in EPR spectra (transitions +7/2 ↔ +5/2, +5/2 ↔ +3/2, …, −3/2 ↔ −5/2, −5/2 ↔ −7/2) yields twelve lines of hyperfine structure (HFS). This is due to two isotopes with non-zero nuclear magnetic moments, ^151^Eu (nuclear spin *I* = 5/2, abundance 47.8%) and ^153^Eu (nuclear spin *I* = 5/2, abundance 52.2%) [49,50]. The HFS is well resolved for the +1/2 ↔ −1/2 central transition (Figure 11), when the direction of an external magnetic field is either parallel with or perpendicular to the *c* axis, exhibiting almost the same spectral features as in KLuS_2_ [28].

It is expected that either one of the regular cation lattice sites or both simultaneously in the KLaS_2_ and KYS_2_ can host dopants similar to the KLuS_2_:Eu [28], where the Eu^2+^ ions were found at both the potassium and lutetium positions (see Table 1). Both cation sites are surrounded by six sulfur anions, creating trigonal antiprisms of D_3d_ point group (see Figure 1).

In order to enhance spectral resolution and avoid forbidden transitions, most of the measurements were carried out at Q-band. All simulation procedures were performed in “Easyspin 4.5.5 toolbox” program [51].

#### 3.4.1. KLaS_2_:Eu

EPR spectra measured in KLaS_2_:Eu at two characteristic orientations of the magnetic field, ***B***||***c*** and ***B***⊥***c*** are shown in Figure 10. In contrast to KLuS_2_ [28] it seems that the Eu^2+^ ions are preferably embedded at one of the available cation positions in the material. Their EPR spectra contain merely all fine transitions allowed by the spin *S* = 7/2 and no artifacts. The Gd^3+^ ions should substitute for the regular La^3+^ ions since KGdS_2_ compounds exists.

Angular dependencies of the Eu^2+^ and Gd3+ resonances of fine transitions in the plane perpendicular to (0001) (Figures S4 and S5 in Supplementary Materials) were simulated [51] by using the spin Hamiltonian, allowed by the D3d symmetry with addition of $b_{2}^{1}O_{2}^{1}$ term [52]:

(5) $$\hat{\text{H}}={\text{β}}_{e}S_{z}gH+b_{2}^{0}O_{2}^{0}+b_{2}^{1}O_{2}^{1}+b_{4}^{0}O_{4}^{0}$$

Here β_e_, *S*_z_, *g*, *H* are the Bohr magneton, electron spin operator, *g* factor (isotropic for the *S* = 7/2), magnetic field, respectively; $b_{2}^{0}$ (axial), $b_{2}^{1}$, $b_{4}^{0}$ (cubic) are crystal field parameters; $O_{2}^{0},O_{2}^{1},O_{4}^{0}$ are the Stevens operators. Terms with the higher order operators, allowed by the *D*_3d_ local symmetry, were neglected, as usually they are much smaller than the terms with $b_{2}^{0}$, $b_{4}^{0}$ components [53]. The angular variations in the (0001) plane show nearly axial symmetry of the corresponding spectra (Figure S16 in Supplementary Materials). Therefore the crystal field parameter $b_{2}^{2}$ was not included in the spin Hamiltonian.

The *g* factors and crystal field parameters $b_{2}^{0},b_{2}^{1},b_{4}^{0}$ were thus determined for both ions and are listed in Table 5. The value of $b_{2}^{1}$ is comparable with $b_{2}^{0}$, clearly proving that the local surroundings of the Eu^2+^ and Gd^3+^ ions do not possess *D*_3d_ symmetry.

**Table 5:** Spin-Hamiltonian parameters of the Eu^2+^/Gd^3+^ ions in the different materials.

Material	KLaS_2_:Eu		KYS_2_:Eu				KLuS_2_:Eu [28]	
**Ion**	Eu^2+^	Gd^3+^	Eu^2+^			Gd^3+^	Eu^2+^	
**Center**			Eu1	Eu2	Eu3		Eu1	Eu2
***g* factor (±0.0005)**	1.9921	1.9917	1.9882	1.9982	2	1.9882	1.992	
$b_{\text{2}}^{\text{0}}$ **(±0.0005 cm^−1^)**	0.0580	0.0395	0.0910	0.0870	0.0820	0.0242	0.1125	0.1018
$b_{2}^{1}$ **(±0.005 cm^−1^)**	−0.030	−0.015	-					
$b_{4}^{0}$ **(±0.0005 cm^−1^)**	2·× 10^−4^	2·× 10^−4^	2·× 10^−4^	2·× 10^−4^	2·× 10^−4^	1.16·× 10^−4^	4	2
**|A_1_(^151^Eu)|, MHz (*B*||*c*)**	87.5	-	87.5			-	89.4	
**|A_2_(^153^Eu)|, MHz (*B*||*c*)**	38.5		38.5				39.75	

In Figure 11 the HFS of the Eu^2+^ +1/2 ↔ −1/2 central transition (***B***||***c***) was almost perfectly approximated by the simulated spectrum [51]. The ^151,153^Eu hyperfine constants along the *c* axis were derived and are listed in Table 5 as well.

The ratio of Eu^2+^ to Gd^3+^ concentrations in the material was nearly six. It was calculated from the corresponding integral line intensities of the spectra.

#### 3.4.2. KYS_2_:Eu and KLuS_2_:Eu

**Figure 12:**
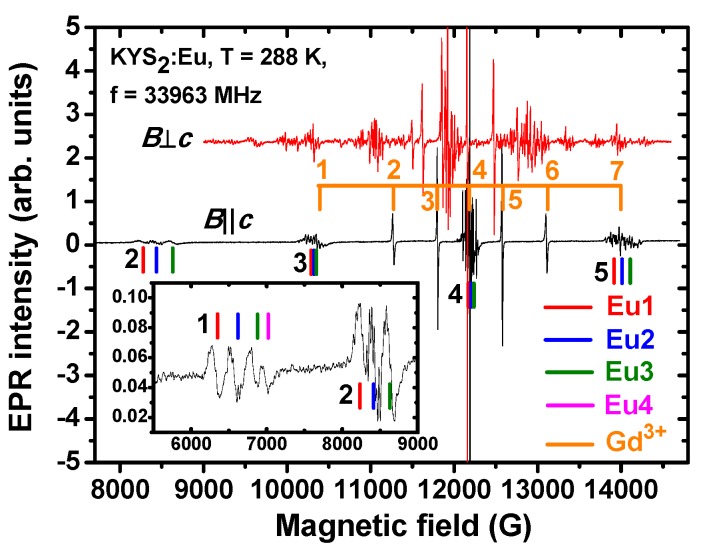
EPR spectra measured in KYS_2_:Eu single crystal at two magnetic field directions ***B***||***c*** and ***B***⊥***c***. The numbers are assigned to particular transitions similar to Figure 10. Inset demonstrates the low field edge of the spectrum where the line segments indicate the transitions produced by four Eu^2+^ centers of almost equal intensity. They are designated as Eu1, Eu2, Eu3 and Eu4.

EPR spectra of the Eu^2+^ and Gd^3+^ ions measured in KYS_2_:Eu are shown in Figure 12 for two characteristic orientations of magnetic field ***B***||***c*** and ***B***⊥***c***.

In contrast to KLaS_2_ EPR spectra in KYS_2_ prove the existence of several distinct positions of the Eu^2+^ ion in the lattice with different strength of crystal field. It can be seen in the low field edge of the Eu^2+^ spectrum at ***B***||***c***. Four and three resonance lines of almost equal intensity corresponding to the −7/2 ↔ −5/2 and −5/2 ↔ −3/2 spin transitions (“1” and “2” in the inset of Figure 12) belong to four Eu^2+^ centers designated as Eu1, Eu2, Eu3 and Eu4. Only the spectral components of the Eu1, Eu2, and Eu3 centers survive in the ***B***||***c*** to ***B***⊥***c*** angular dependence (Figure S7 in Supplementary Materials) and were analyzed in detail. Similar to the KLaS_2_:Eu, the Gd^3+^ ions were assumed to substitute for the Y^3+^ ions. Angular dependencies of the corresponding fine components are in Figure S8 in the Supplementary Materials.

The *g* factors, axial and cubic crystal field terms of both paramagnetic species and HF constants (for Eu^2+^ only) were determined following the procedure applied to the KLaS_2_:Eu above. They are listed in Table 5. Unlike KLaS_2_:Eu, the crystal field parameter $b_{2}^{1}$ is much smaller than $b_{2}^{0}$ and was neglected therefore, proving that the local surroundings of the centers are only slightly perturbed. Angular variations of the Eu^2+^ and Gd^3+^ spectra (Figure S9 of supplementary materials) in the (0001) plane exhibit nearly axial symmetry similar as in KLuS_2_ [28] and KLaS_2_. The concentration ratio *n*(Eu^2+^)/*n*(Gd^3+^) was about 1.25. Even with such a small ratio there are four centers of the Eu^2+^ as compared to the KLaS_2_:Eu, where the Eu^2+^ ions occupy only one site. Thus, the role of the Gd^3+^ ions does not seem critical for the Eu^2+^ incorporation in the KYS_2_:Eu.

It should be noted that no Gd impurity was found in the X-ray fluorescence spectra of KYS_2_:Eu, whereas Eu in 0.05% concentration was still detectable. When combined with the EPR measurements, this observation suggests that the majority of Eu ions in the KYS_2_ sample are presented in the form of non-paramagnetic Eu^3+^ and the actual concentration of Eu^2+^ is very low, comparable with the concentration of background Gd impurity. Probably, a similar situation takes place in other ALnS_2_:Eu sulfides, which can be corroborated by very high emission intensity in KLuS_2_:Eu doped with only 0.002% Eu [27].

The Eu^2+^ ions at two cation positions in the KLuS_2_:Eu [28] and KYS_2_:Eu can reasonably be ascribed to the lattice sites in the way that the higher $b_{2}^{0}$ value corresponds to the smaller Ln–S distance, whereas the lower one to the larger K–S distance. The Eu1 and Eu2 centers in the KYS_2_:Eu are supposed to be created by substitution of the Eu^2+^ for the Y^3+^ ions with regular and somehow perturbed ligand surroundings, respectively. Similarly, the Eu3 and Eu4 centers were assigned to the K^+^ sites.

The most profound difference among KLnS_2_ (Ln = La, Lu, Y) is between *d*_La–S_ and *d*_Lu/Y–S_ distances (0.197, 0.153 Å, respectively, Table 2) so the crystal field strengths of the trivalent sites should vary much more. The difference between K–S distances in the mentioned materials is in the range 0.020–0.088 Å (from Table 2, *d*_K–S_(KYS_2_) − *d*_K–S_(KLuS_2_) = 3.174 Å − 3.154 Å = 0.020 Å and *d*_K–S_(KLaS_2_) − *d*_K–S_(KLuS_2_) = 3.242 Å − 3.154 Å = 0.088 Å), assuming slight deviations between the local crystal field strengths. Therefore, the Eu^2+^ ion most probably occupies namely the La^3+^ regular lattice site in KLaS_2_. Its axial constant $b_{2}^{0}$ in the KLaS_2_ is almost two times lower than that in the KLnS_2_:Eu (Ln = Lu, Y). The mechanisms of charge compensation in KLaS_2_:Eu for the Eu^2+^ at the trivalent site thus can be either $2{\text{Eu}}_{\text{La}}^{-}+{\text{La}}_{\text{K}}^{\text{2+}}$ or ${\text{Eu}}_{\text{La}}^{-}+{\text{La}}_{\text{K}}^{\text{2+}}+K_{\text{La}}^{-}$ (*V_k_* denotes the potassium vacancy). The second charge compensation scheme is less likely, since concentration of potassium vacancies would then need to be similar to the Eu^2+^ ions concentration. Such a great number of vacancies might cause very strong perturbation of the Eu^2+^ local environment, significantly reducing the local trigonal symmetry. This impact on the local ligands should be detectable as the presence of anisotropy in the corresponding EPR spectra in the (0001) rotation plane. The first compensation mechanism $2{\text{Eu}}_{\text{La}}^{-}+{\text{La}}_{\text{K}}^{\text{2+}}$ corresponds to slight distortions of the trigonal antiprism (see Figure 1) since the presence of the antisite defects nearby (${\text{La}}_{\text{K}}^{\text{2+}}$) could hardly have a strong influence on the local Eu^2+^ surroundings as their concentration is two times lower than the concentration of the Eu^2+^ dopants. Most probably, the antisite defects are responsible for the mentioned local symmetry break in the KLaS_2_:Eu.

The characteristic emission lines of the Eu^3+^ ions, which are “invisible” for EPR, were observed in the luminescence spectra of all studied sulfides except for KLaS_2_:Eu in the temperature range 8–200 K (Section 3.3). We measured temperature dependencies of Eu^2+^ EPR spectra in KYS_2_ and KLuS_2_in the temperature range 20–298 K (Figures S10 and S11 in Supplementary Materials). No significant changes in the spectra occurred while cooling the samples to 40 K. Below this temperature the spectra become saturated due to long spin-lattice relaxation times. The ratio of resonance line intensities of at least two clearly visible spectral components originating from Eu^2+^ centers was constant in the temperature range 40–298 K.

This is in a good agreement with the TD of RL data for KLuS_2_:Eu (0.05%). Eu^2+^ was claimed to occupy three different sites in the KLuS_2_ structure [28], namely the K^+^ site, Lu^3+^ site and defect-based sites (see also above). These sites provide slightly different emissions which can be obtained by decomposition of the spectra into three Gaussians. Therefore we decomposed the measured RL spectra at each temperature (see details in [28]). We assumed that the positions (in eV) of each of three Gaussian components are temperature independent and therefore only band widths (expressed as full width at half maximum (FWHM) and amplitudes were varied in the fitting process. The product of *i*-th band amplitude and *i*-th band width provides the information about intensity released by the *i*-th band. These products are indeed more or less constant, see Figure S12 in the Supplementary Material, well matching the above-mentioned EPR results. Thus, the Eu^3+^ ions exist in the materials initially along with the Eu^2+^ ions and are not created due to the charge transfer between the Eu^2+^ centers.

### 3.5. Energy Diagram of Lanthanide Levels in KLuS_2_

**Figure 13:**
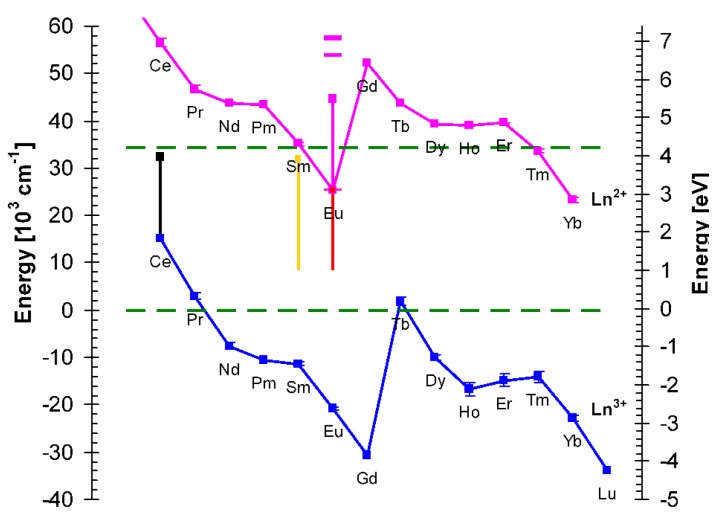
The proposed lanthanide energy level scheme in KLuS_2_ at 77 K, description in text.

Figure 13 shows the most probable energy diagram of lanthanide energy levels in KLuS_2_ host at 77 K constructed from the measured luminescence properties. Low temperature was chosen to interpret the Eu^3+^ emission, which occurs only at lower temperatures. While discussing the energy levels of europium, we assume that both Eu^2+^ and Eu^3+^ ions occupy the Lu^3+^ position in the KLuS_2_ structure. The band gap of KLuS_2_ at 77K estimated from photoluminescence excitation spectrum is at *ca*. 291 nm (4.26 eV) and it corresponds to the distance between the top of the valence band and the bottom of the conduction band of the host lattice (the horizontal dashed lines in Figure 13). From the position of the Eu^3+^ CT band in PLE spectra at 396 nm (3.13 eV, see Figure 8), we can locate the Eu^2+^ 4f ground state to the energy diagram, following the procedure of Dorenbos [54], according to which the CT process starts from the top of the valence band and the final state is the ground state of the divalent lanthanide. We also note here that the Eu^3+^ CT band position and shape in the PLE spectra are practically identical for Eu-doped KLuS_2_, KYS_2_, KGdS_2_, RbLuS_2_ and NaLuS_2_ (not shown here) at 77 K. Knowledge of the Eu^2+^ ground state position allows us to approximately determine the position of the Eu^3+^ ground state as well. Energy difference ΔE(Eu) between the 4f^6^ ground state of Eu^3+^ and the 4f^7^ ground state of Eu^2+^ is reported to reflect the type of anions in the compound and was very roughly estimated to be ≈5.7 eV in the ternary sulfide host [55] (namely in CaGa_2_S_4_ [7]). We would like to stress that this is a very rough approximation and can only be used for qualitative description. Following such an approach [55], the ground state of Eu^3+^ in KLuS_2_ at 77 K was shown to be deeply inside the valence band (Figure 13), possibly even under the valence band. Similarly, the Eu^3+^^5^*D*_0_ excited state seems to lie inside the valence band as well. A very approximate valence band width of 4 eV can be derived using [56] where the electronic structure of RbLnSe_2_ was calculated, which is isostructural with ALnS_2_ sulfides. However, the characteristic intense Eu^3+^ emissions from ^5^*D*_0_ level to ^7^*F*_x_ levels were clearly observed in the KLuS_2_ host at low temperature (see Figure 9), which means that the energy from the CT state (4f ground state of Eu^2+^) is transferred, probably via intersystem crossing, to an excited state of Eu^3+^. This is a common situation for (Eu^3+^) [54]. Interestingly, the energy diagram of lanthanide energy levels in CaGa_2_S_4_ [7] shows that the CT band of Eu^3+^ is predicted even below 2 eV. Such low values in practice imply that Eu^3+^ is not stable in CaGa_2_S_4_ and therefore Eu^2+^ is formed during synthesis [48].

Another possibility of how to estimate the Eu^3+^ CT transition position in the forbidden gap is to use the known value of Sm^3+^ CT in KLuS_2_ which is situated at 313 nm (3.96 eV, vertical dots in Figure 13) [30]. According to Dorenbos [57], the energy difference between CT Sm^3+^ and CT Eu^3+^ is equal to *ca*. 9800 cm^−1^ (1.22 eV), which locates the Eu^3+^ CT in KLuS_2_ at 2.75 eV. This is not far from the experimentally obtained value 3.13 eV. The error is assumed to be systematic for each lanthanide and on the order of 0.5 eV [58]. It needs to be mentioned here that a similar lanthanide energy level scheme for a compound with comparable band gap, namely GaN (band gap 3.42 eV), was published [55,59]. Dorenbos had already published numerous papers connected to such energy diagrams for various compounds, for example YPO_4_ [54], Y_2_O_3_, CaBPO_5_, KCl [60], CaF_2_ [54], Al*_x_*Ga_1 - *x*_N [55] and therefore following his procedure was also considered applicable for our KLuS_2_ ternary sulfide.

From what was said above a crucial question arises: What is the cause of Eu^3+^ quenching in KLuS_2_? We believe that an explanation will also be valid for other Eu-doped ternary sulfides, in which we observed Eu^3+^ emission at low temperature, those being KGdS_2_, KYS_2_, RbLuS_2_ and NaLuS_2_. First, it is rather unlikely that classical thermal quenching (ergo return of the electron from the excited state of Eu^3+^ to its ground state via phonon interaction without any radiation) would be responsible for observed Eu^3+^ vanishing. In [61] it is shown that the temperatures of thermal quenching for Eu^3+^ emission (when excited via Eu^3+^ CT band) are very much above RT even in oxysulfides. Secondly, thermally induced ionization of the Eu^3+^ exited state to the conduction band of host is completely unfeasible as this state lies within the valence band of the host. Ionization to any state within the valence band is excluded as all the states should be occupied by electrons. Based on the work of Blasse [48] it appears that a possible source of Eu^3+^ quenching might by the crossing of the Eu^3+^ excited and ground state parabolas with the parabola representing the Eu^3+^ CT state (see Figure 1 in [48]). At low temperature Eu^3+^ emission is observed. With an increasing temperature system in the Eu^3+^ excited state ^5^*D*_0_ can acquire thermal energy (≈370 meV) sufficient to reach the crossing point with the CT state parabola, in which case no light emission would be observed.

Moreover we are aware that showing energy diagram as depicted in Figure 13 cannot explain every Eu feature we have investigated. Eu^2+^ 4f-5d absorption band in KLuS_2_ at low temperature peaks at 394 nm and emission 5d-4f at 520 nm (see Figure 8). Taking into account both the position of the Eu^2+^ ground state 3.13 eV above the top of the valence band and the diagram from Figure 13, the Eu^2+^ excited state would have to be buried in the conduction band of the KLuS_2_ host. It implies that the Eu^2+^ center would be ionized at any temperature. However, this is not observed. From Eu^2+^ 5d-4f photoluminescence decay time measurements we know that the decay time shortening starts around 480 K (see Figure 6c). Baran *et al.*, investigated binding energies of europium in β-Ca_2_SiO_4_ doped (purposely) by both Eu^2+^ and Eu^3+^ ions [62]. They proposed that both conduction and valence bands can bend (see Figure 13 in [62]). The band bending occurs in the vicinity of a certain defect and two Eu^3+^ ions. It has a local character, because the defect and two Eu^3+^ ions do not create long range Coulomb potential. Possibly, similar local band bending can appear in the Eu-doped KLuS_2_, promoting a location of the Eu^2+^ excited state under the bottom of the conduction band. The latest approach of energy level modeling of lanthanide materials is published in [63]. Nevertheless, more experimental work, both optical and paramagnetic, will definitely have to be carried out in the future to complete an explanation of all the observed features.

### 3.6. CIE Coordinates

**Figure 14:**
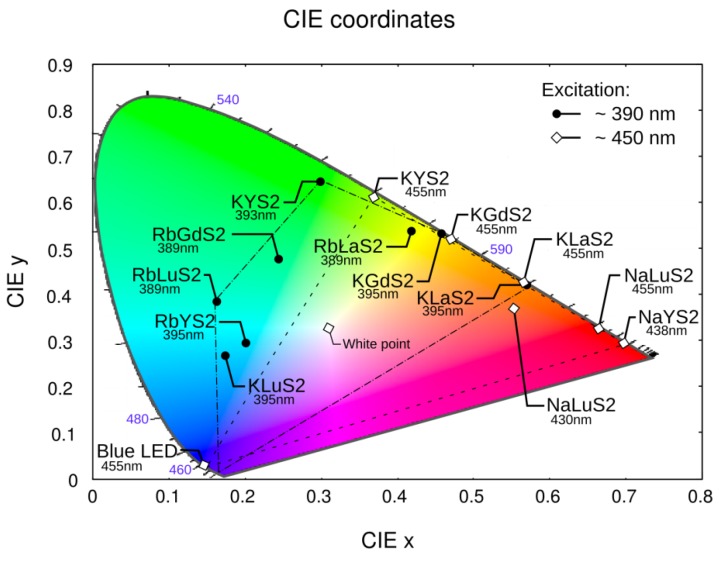
Commission Internationale de I’Eclairage (CIE) 1931 color coordinates calculated for samples under ~390 nm excitation (dark points, labeled inside) and ~450 nm excitation (empty diamonds, labeled outside)—Actual excitation is written below each sample label. Blue light emitting diode (LED) at 460 nm added for comparison. Lines denote colors available by mixing multiple materials under 390 nm and 455 nm excitation, respectively.

CIE 1931 coordinates were calculated for the presented samples under different excitations, see Figure 14. Dashed (exc. 390 nm) and dash-and-dot (excitation 455 nm) lines show colors available by mixing the emission spectra of the samples—A large area of visible color space is covered, which outlines a great potential in solid-state lighting applications. KLuS_2_, RbYS_2_, RbLuS_2_, under 395 nm excitation, provide a good opportunity for tuning white correlated color temperature (CCT). The same is valid for NaLuS_2_ and KLaS_2_ under 455 nm excitation, where the red color produced could find its application also in improving the color rendering index (CRI) of state-of-the-art materials (e.g., YAG:Ce with 455 nm blue LED source, where mainly blue and yellow light is present).

**Figure 15:**
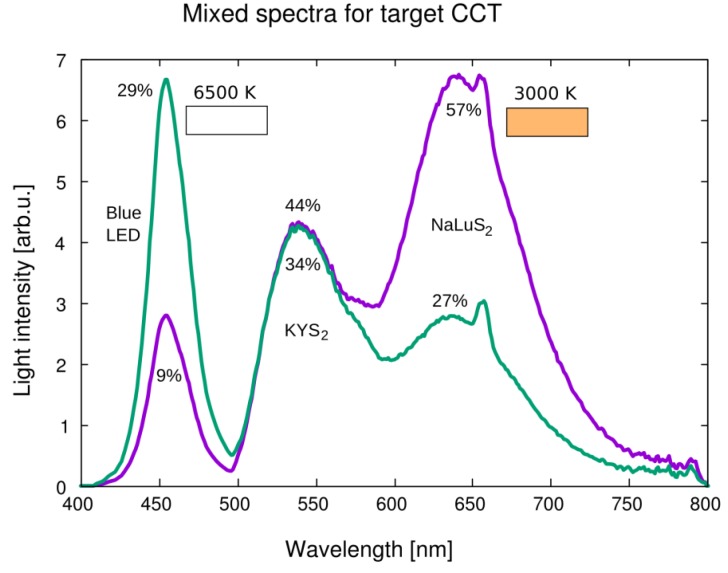
Spectral profile obtained by combination of three spectra (blue LED source, KYS_2_ and NaLuS_2_) to obtain 3000 K (9%, 34%, 57%) and 6500 K (29%, 44%, 27%) light with 455 nm excitation source. Approximate resulting white light colors are demonstrated in color boxes. CCT: correlated color temperature.

To demonstrate the potential of studied materials, combined spectra were calculated for 455 nm excitation and target CCT of 3000 K and 6500 K, respectively, using blue LED source, KYS_2_ and NaLuS_2_ as building blocks (see Figure 15). Composition of the spectra was calculated using an optimization routine. Using other presented materials and their different active volume, a large area of color space is available for composed light devices (denoted by lines in Figure 14). A slight difference will be present in reality, because we use the emission spectrum of the source, while in the real applications the spectrum needed is an emission spectrum of the source after passing through the light device to the detector (*i.e.*, after light absorption).

## 4. Conclusions

The current work presents a new family of optical materials, namely Eu-doped ternary sulfides ALnS_2_ (A = Na, K, Rb; Ln = La, Gd, Lu, Y), as potentially interesting for solid state lighting and X-ray phosphors applications. A set of single-crystalline platelets of Eu-doped ALnS_2_ were successfully synthesized. Interesting dependence of Eu^2+^ 5d-4f emission energy, covering a range from 498 nm (RbLuS_2_:Eu) to 779 nm (NaGdS_2_:Eu), on structural parameters was found and was explained by crystal field theory. Temperature stability of Eu^2+^ decay times, needed for white LED applications, was confirmed mainly for ALuS_2_:Eu. In particular, decay time values at 497 K still reach 80%, 70% and 45% of their low-temperature limits for KLuS_2_, RbLuS_2_ and NaLuS_2_, respectively. Eu^2+^-doped KLaS_2_, on the other hand, suffers from strong quenching already slightly above room temperature. All the decay time temperature dependencies were fitted by a phenomenological model and the list of best fit parameters was summarized. EPR revealed that Eu^2+^ ions occupy only a single, three or four different sites in KLaS_2_, KLuS_2_, and KYS_2_, respectively, and a charge compensation mechanism $2{\text{Eu}}_{\text{La}}^{-}+{\text{La}}_{\text{K}}^{\text{2+}}$ for Eu^2+^ in La^3+^ position in KLaS_2_ was suggested. Characteristic ^5^*D*_0_-^7^*F_x_* emission lines in the 570–730 nm spectral region attributed to Eu^3+^ appeared under X-ray, UV and VIS excitation at low temperatures (below 200 K) in Eu-doped KLuS_2_, KYS_2_, KGdS_2_, RbLuS_2_ and NaLuS_2_. These lines are completely absent in Eu-doped KLaS_2_. By means of EPR, it was concluded that the Eu^3+^ ions do not appear in the sulfide at low temperatures because of the charge transfer process, but initially exist there at room temperature as well. At low temperatures, excitation spectra associated with the Eu^3+^ emission show a broad intense band peaking at 393 nm. This band was assigned to Eu^3+^ charge transfer state (CT). Position of this Eu^3+^ CT band and known energy difference ΔE(Eu) between the 4f^6^ ground state of Eu^3+^ and the 4f^7^ ground state of Eu^2+^, which is reported to be ≈5.7 eV in the ternary sulfide host, were used to construct the most probable energy diagram of lanthanide energy levels in the KLuS_2_ host. CIE coordinates of all the studied samples were calculated for ~390 nm and ~450 nm excitations. Due to elevated density (5.2 g/cm^3^ for RbLuS_2_), effective atomic numbers (61.4 for RbLuS_2_) and high light output (35,000 ph/MeV for KLuS_2_:Eu (0.05%)), these materials can be applied as X-ray phosphors for γ/X-ray detection. Furthermore, thanks to the presence of a broad emission band of Eu^2+^, whose position can be tuned by different chemical composition, suitable location of absorption bands in the 350–450 nm region, high thermal stability of Eu^2+^ emission and the possibility to produce ALnS_2_ in the form of transparent single-crystalline platelets, Eu-doped ALnS_2_ as such are also promising candidates for white LED solid-state lighting.

## Supplementary Materials

The following are available online at www.mdpi.com/1996-1944/8/10/5348/s1.
