# Erratum: Jarý, V.; *et al.* Optical, Structural and Paramagnetic Properties of Eu-Doped Ternary Sulfides ALnS_2_ (A = Na, K, Rb; Ln = La, Gd, Lu, Y). *Materials* 2015, *8*, 6978–6998

**DOI:** 10.3390/ma8115420

**Published:** 2015-11-13

**Authors:** Materials Editorial Office

**Affiliations:** MDPI AG, Klybeckstrasse 64, CH-4057 Basel, Switzerland; materials@mdpi.com

The *Materials* Editorial Office wishes to make the following erratum to this paper [1]. There is a layout mistake in Table 5 in the original published version. The correct Table 5 is shown below:

**Table 5:** Spin-Hamiltonian parameters of the Eu^2+^/Gd^3+^ ions in the different materials.

Material	KLaS_2_:Eu		KYS_2_:Eu				KLuS_2_:Eu [28]	
**Ion**	Eu^2+^	Gd^3+^	Eu^2+^			Gd^3+^	Eu^2+^	
**Center**			Eu1	Eu2	Eu3		Eu1	Eu2
***g* factor (±0.0005)**	1.9921	1.9917	1.9882	1.9982	2	1.9882	1.992	
$b_{\text{2}}^{\text{0}}$ **(±0.0005 cm^−1^)**	0.0580	0.0395	0.0910	0.0870	0.0820	0.0242	0.1125	0.1018
$b_{2}^{1}$ **(±0.005 cm^−1^)**	−0.030	−0.015	-					
$b_{4}^{0}$ **(±0.0005 cm^−1^)**	2 × 10^−4^	2 × 10^−4^	2 × 10^−4^	2 × 10^−4^	2 × 10^−4^	1.16 × 10^−4^	4	2
**|A_1_(^151^Eu)|, MHz (*B*||*c*)**	87.5	-	87.5			-	89.4	
**|A_2_(^153^Eu)|, MHz (*B*||*c*)**	38.5		38.5				39.75	

We would like to apologize for any inconvenience caused to the readers by this mistake.
