# Supplementary material for: Optical, Structural and Paramagnetic Properties of Eu-Doped Ternary Sulfides ALnS2 (A = Na, K, Rb; Ln = La, Gd, Lu, Y)
Source: Materials (Basel). 2015 Oct 13;8(10):6978–98. doi: 10.3390/ma8105348 (PMC5455392; doi:10.3390/ma8105348)
Supplement: Supplementary file 1 [file materials-08-05348-s001.pdf]

Supplementary Information

Dependence of Eu^{2+} Emission Energy on the Host Structure *c*/*a* Ratio

The Eu^{2+} emission wavelength offset was observed in the row of ternary sulfides with common chemical formulae $ALnS_2$:Eu (A = Na, K, Rb; Ln = La, Gd, Y, Lu). The wavelengths were converted to the emission energy and its dependence on *c*/*a* ratio for each the material (*c*, *a* are the dimensions of the unit cell) is shown in Figure S1.

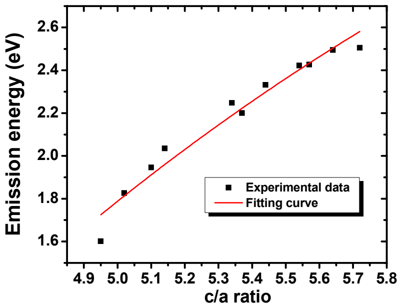

Figure S1. Dependence of Eu^{2+} emission energy on the host structure *c*/*a* ratio for each material studied.

The Eu^{2+} emission energy is given by [S1]

$$E_{\text{em}} = E_{\text{free}} - D - \Delta S \quad \text{(S1)}$$

where E_{free} is the free ion energy, D and ΔS are the redshift and Stokes shift in chosen compound.

For Ce^{3+} the redshift would be [S2–S5]:

$$D^{\text{Ce}} = E_{\text{c}}^{\text{Ce}} + mE_{\text{cfs}}^{\text{Ce}} - 0.234\ \text{eV} \quad \text{(S2)}$$

where E_c and E_{cfs} are the centroid shift and crystal field splitting of the 5d excited state, m is the constant, representing a contribution of the crystal field splitting to the redshift. Since the Ce^{3+} redshift, Stokes shift, centroid shift and crystal field splitting must be linearly related to those of Eu^{2+} [S1], Equation (S2) could be slightly modified by inserting some proportionality constant b. Taking this into account and expressing the centroid shift in the first approximation after [S2,S6,S7], the emission energy in Equation (S1) for Eu^{2+} in sixfold coordination of S^{2-} (D_{3d} point symmetry group) becomes:

$$E_{\text{em}} = E_{\text{free}} + 0.234b - \Delta S - \frac{e^2}{4\pi\varepsilon_0}\frac{\left(\langle r^2\rangle_{5\text{d}} - \langle r^2\rangle_{4\text{f}}\right)}{R^6}\sum_{i=1}^{6}\alpha_i - mE_{\text{cfs}} \quad \text{(S3)}$$

where r is the coordinate of an electron in either the 5d or 4f orbital, α_i is the polarizability of the i-th ligand at a distance R from the Eu^{2+} ions, e is the elementary charge, and ε_0 is the permittivity of vacuum.

The splitting of the excited state $4f^6 5d^1(^2D)$ by the local crystal field is schematically shown in Figure S2.

The local symmetry of either M or Ln perturbed octahedra in the materials is D_{3d} (Figure S2a). Using the method of descending symmetry [S8], one can find the Eu^{2+} ion excited state splitting due to ligand field. Initially undistorted octahedron has the O_h local symmetry, which splits the nd^1 outer shell into T_{2g} (ground level, triply degenerated) and E_g (doubly degenerated) (see e.g., [S2]). When the local symmetry is reduced, additional splitting of the levels appears. The T_{2g} and E_g levels are decomposed, as it is shown in Figure S2b, into irreducible representations of the D_{3d} point group [S8]. The electric dipole transition 5d-4f occurs between the lowest level ($E_g(d_{xz}, d_{yz})$) of the excited state $4f^6 5d^1(^2D)$ after removing degeneracy by the crystal field and the ground state $4f^7(^8S_{7/2})$. Due to variation of the local crystal field strength depending on the lattice type, the quantity δ (the energy separation between the ^2D state shifted by the centroid shift, and the $E_g(d_{xz}, d_{yz})$ additionally lowered by the Stokes shift) also changes accordingly. There might also be additional weak splitting

of the lowest $E_g(d_{xz}, d_{yz})$ level caused by slight local distortions of the crystal field and/or influence of more distant ligands, having hexagonal symmetry (see Figure 1 in the main text). Using the crystal field theory (for details see e.g., [S9]), with some simplifications, the energy E_{cfs} of the lowest $E_g(d_{xz}, d_{yz})$ (Figure S2) could be the following:

$$E_{\text{cfs}} = \left(\frac{A}{\left(1+\left(\xi \, c/a\right)^2\right)^{3/2}} - \frac{B}{\left(1+\left(\xi \, c/a\right)^2\right)^{5/2}} \right) \tag{S4}$$

where the M,Ln-S distance is $R = \left(\left(c/12 \right)^2 + a^2/3 \right)^{1/2}$, assuming for simplicity $c_{\text{M,Ln}} = \left(c_{\text{M}} + c_{\text{Ln}}\right)/2 = c/6$ (see Figure 1 in the main text), $\xi^2 = 1/48 \approx 0.0208$. A and B are the coefficients accounting for the Ln ion charge and expectation values of the r electron coordinates $\langle r^2 \rangle$ and $\langle r^4 \rangle$ in the Eu^{2+} 5d orbital, respectively.

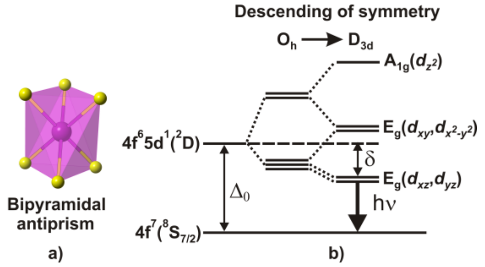

Figure S2. (**a**) Local sulphur surrounding (six S^{2-} ions) of either mono or trivalent cation site (in the center), having the trigonal symmetry of bipyramidal antiprism (D_{3d} symmetry group). (**b**) Schematic energy level diagram (ground and first excited states) of the Eu^{2+} ion; the splitting of the excited level $4f^6 5d^1(^2D)$ due to the local crystal field (a) is shown. Δ_0 is the energy separation between the ^2D excited state after the centroid shift and ground state; δ accounts for the Stokes shift of the lowest $E_g(d_{xz}, d_{yz})$ level as well.

The emission energy (Equation (S3)) then transforms as:

$$\begin{aligned} E_{\text{em}} &= \Delta - \frac{A'}{\left(1+\left(\xi \, c/a\right)^2\right)^{3/2}} + \frac{B'}{\left(1+\left(\xi \, c/a\right)^2\right)^{5/2}} \\ A' &= mA \\ B' &= mB - C/\langle R \rangle \\ C &= \frac{e^2}{4\pi\varepsilon_0}\left(\langle r^2 \rangle_{5d} - \langle r^2 \rangle_{4f} \right) \sum_{i=1}^{6} \alpha_i \end{aligned} \tag{S5}$$

here $\Delta = E_{\text{free}} + 0.234b - \langle \Delta S \rangle$, we assume the Stokes shift on average to be constant for the row of materials studied; $\langle R \rangle = \sum_{i=1}^{N} R_i \Big/ N$, $N = 11$ is the number of studied compounds (Figure S1).

Instead of four parameters Δ, A, B and C to vary, in the first approximation we thus obtain only three Δ, A' and B'. It does not much influence the precision of the fit, since the more the parameters vary in the linear combination, the less accurate are the values they get.

Fitting the curve calculated from Equation (S5) to the experimental dependence in Figure S1, the following parameters were obtained: Δ = 4.7 ± 0.2 eV, $A' = -4.4 \pm 1$, $B' = -14.4 \pm 2$. Due to simplifications introduced by algebraic manipulations the uncertainties of determined A' and B' constants may be quite large. However, the important question about the Eu^{2+} emission wavelength shift has been semi-quantitatively solved.

Luminescence and EPR Experiment—Additional Data

As an example, four normalized decay curves of KLnS2:Eu (Ln = Lu, Y, Gd, La; 0.05% Eu) are shown in Figure S3. Interestingly, their signal-to-background ratio improves in the KLuS2:Eu-KYS2:Eu-KGdS2:Eu-KLaS2:Eu series, which may be related to processes of the excited state ionization of the Eu^{2+} activator, at least in the KGdS2, KLaS2 hosts, see the main document.

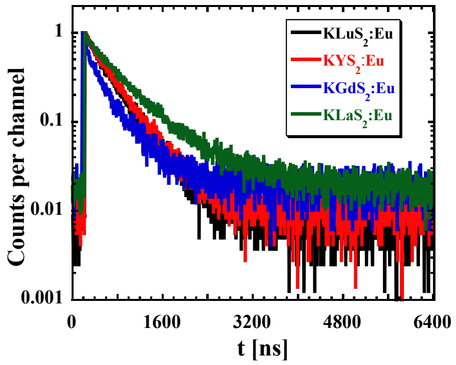

Figure S3. RT Normalized decay curves related to the Eu^{2+} 5d-4f transition in KLnS2:Eu (Ln = Lu, Y, Gd, La); Excitation and emission wavelengths taken from Table 3 in the main document; data of KLuS2:Eu after [S10].

In order to obtain the spin-Hamiltonian parameters (see main body of the article) for the Eu^{2+} and Gd^{3+} ions (uncontrolled impurity) in the KLaS2:Eu, the simulated in "Easyspin 4.5.5 toolbox" program [S11] curves were fitted to the angular variations of the corresponding resonance magnetic fields obtained by the **B**||**c** → **B**⊥**c** rotation of a sample (see Figures S4 and S5).

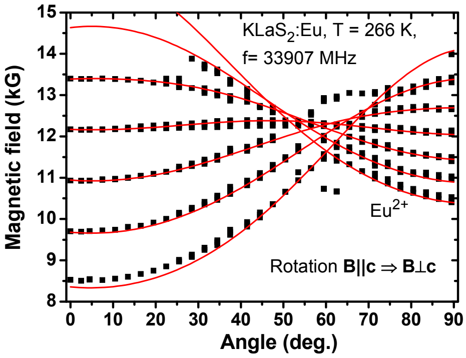

Figure S4. Angular dependence of the resonance lines produced by the Eu^{2+} ions measured in KLaS2:Eu single crystal. Dots represent experimental data and solid lines are the fitting curves simulated in "Esyspin 4.5.5 toolbox" program [S11]. f is the microwave frequency.

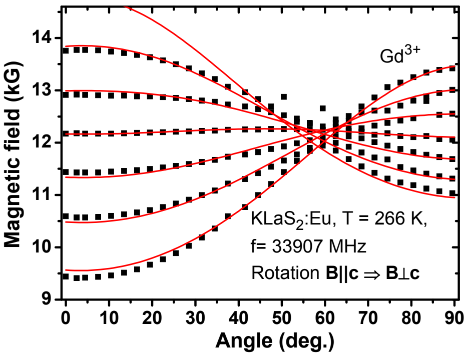

Figure S5. Angular dependence of the resonance lines produced by the Gd^{3+} ions measured in KLaS2:Eu single crystal. Dots represent experimental data and solid lines are the simulated fitting curves [S11].

The simulated curves show good agreement with experimental data. However, the broadening of the Eu^{2+} hyperfine lines and intermixing of spectral components originating from both the ions are, mostly, responsible for the local worsening of the fit. Besides, as the sample was of a platelet shape, one can expect some slight inhomogenity of its structure on the edges and surface of the sample (gradual tensions could exist). The rather indented edges do not allow precise determination of the direction in the rotational plane (0001), to take it as a reference point when measuring the angular variations in a perpendicular plane. The scarce deviation from axial symmetry in the (0001) plane (Figure S6) may cause unexpected shifts of the resonance lines as well.

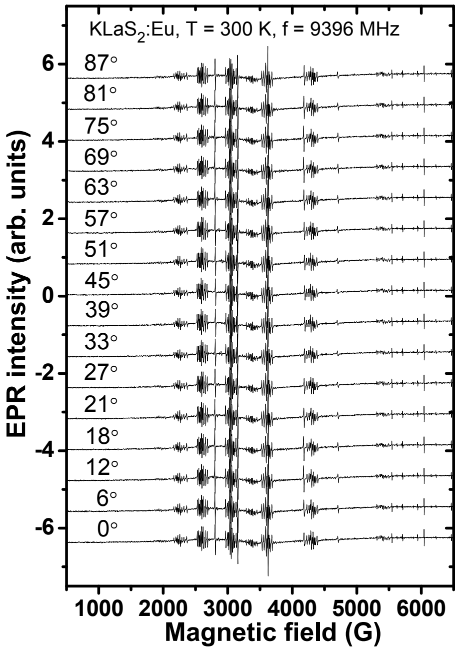

Figure S6. EPR spectra measured in $KLaS_2$:Eu single crystal at the specific magnetic field orientations (assigned with the angle of rotation) in the (0001) rotational plane.

By analogy to the $KLaS_2$:Eu, the same procedure of finding spin-Hamiltonian parameters (Table 5 in the main document) is suitable for KYS_2:Eu. Angular dependences of the Eu^{2+} and Gd^{3+} ions are presented in Figures S7 and S8. The general discrepancy between the materials is that in KYS_2 the EPR lines from four Eu^{2+} centers could be resloved in spectra and only three of them could be treated separately in the angular dependence. In contrast to the KYS_2, in the previous work [S12] we reported the presence of three Eu^{2+} centers created by the substitution of the Eu^{2+} ions for both K and Lu regular lattice cations and the perturbed one.

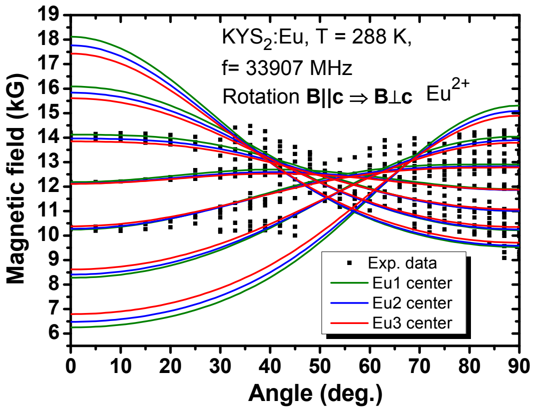

Figure S7. Angular dependencies of the resonance lines produced by the Eu^{2+} centers measured in KYS_2:Eu single crystal. Dots represent experimental data and solid lines are the simulated fitting curves [S11].

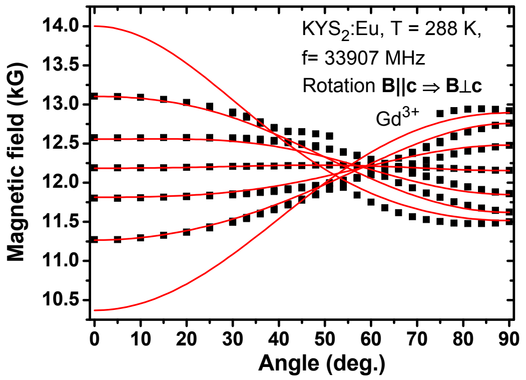

Figure S8. Angular dependence of the resonance lines produced by the Gd^{3+} ions measured in KYS_2:Eu single crystal. Dots represent experimental data and solid lines are the simulated fitting curves [S11].

Although the fit is not perfect (see Figures S7 and S8), the slight disagreement between the fitting curves and experimental data most probably is due to the same reasons as in the case of $KLaS_2$:Eu (see above). The angular dependence in the (0001) plane is in Figure S9. It shows almost axial symmetry similarly to the $KLaS_2$.

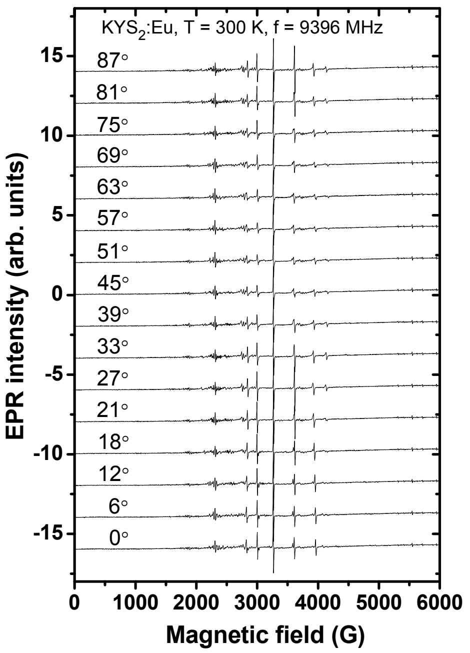

Figure S9. EPR spectra measured in KYS_2:Eu single crystal at the chosen magnetic field directions (spectra assigned with the angle of rotation) in the (0001) rotational plane.

Below 160 K, the light emission from the Eu^{3+} ions occurred in all the sulfides studied except the $KLaS_2$ (see main text of the article). Since they are not paramagnetic, the direct EPR observation is impossible. However, to clarify if some charge transformation ($Eu^{2+} \rightarrow Eu^{3+}$) when lowering the sample temperature indeed took place is possible. With this aim the temperature dependencies of the Eu^{2+} EPR spectra in the KYS_2 (Figure S10, for better visualization, the Gd^{3+} resonances were mostly extracted from the spectra) and $KLuS_2$ (Figure S11) were performed. It is noteworthy that the spectra do not demonstrate signal intensity fading with decreasing temperature. Obversely, the signal-to-noise ratio increases due to elongation of the spin-relaxation time till saturation (approximately at 20 and 50 K for the KYS_2 and $KLuS_2$, respectively). No changes to the intensity distribution between spectral components originating from different Eu^{2+} centers in both the materials was observed as well. This indicates that the $Eu^{2+} \rightarrow Eu^{3+}$ charge transformation does not exist, but the Eu^{3+} ions are initially presented in the materials.

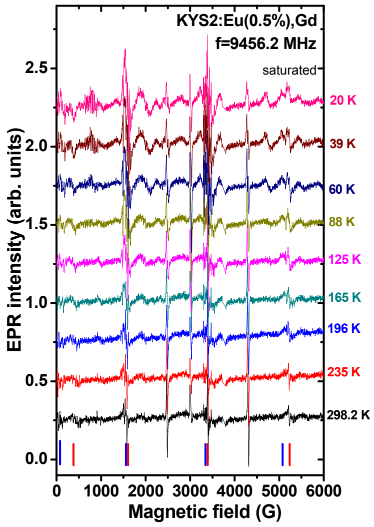

Figure S10. EPR spectra measured in KYS_2:Eu single crystal at the chosen temperatures, distinguished by color accordingly. Blue and red vertical line segments are associated with two clearly visible fine transitions, originating from the two Eu^{2+} centers.

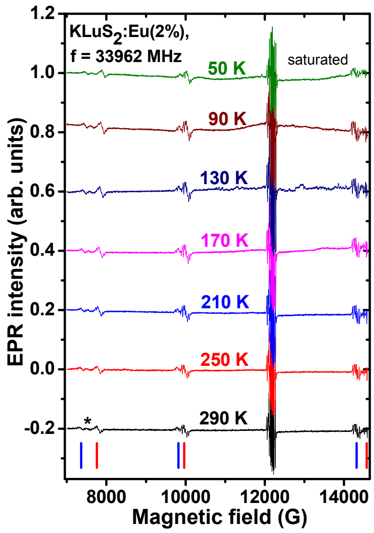

Figure S11. EPR spectra measured in $KLuS_2$:Eu single crystal at the chosen temperatures, distinguished by color accordingly. Blue and red vertical line segments are associated with two clearly visible fine transitions, originating from the two Eu^{2+} centers.

Figure S12 gives EPR and luminescence results together showing that emission intensity released by Eu^{2+} occupying both K^+ and Lu^{3+} sites in $KLuS_2$ is temperature independent.

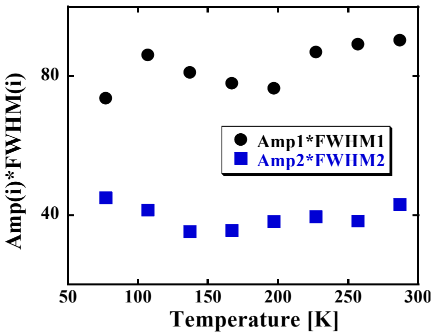

Figure S12. Temperature dependence of the products of amplitudes and band widths of Gaussians components for emission spectra of Eu^{2+}-doped $KLuS_2$ (see the main document for details).

References

S1. Dorenbos, P. Relation between Eu^{2+} and Ce^{3+} f ↔ d-transition energies in inorganic compounds. *J. Phys. Condens. Matter* **2003**, *15*, 4797–4807.

S2. Dorenbos, P. 5d-level energies of Ce^{3+} and the crystalline environment. I. Fluoride compounds. *Phys. Rev. B* **2000**, *62*, 15640–15649.

S3. Dorenbos, P. 5d-level energies of Ce^{3+} and the crystalline environment. II. Chloride, bromide, and iodide compounds. *Phys. Rev. B* **2000**, *62*, 15650–15659.

S4. Dorenbos, P. 5d-level energies of Ce^{3+} and the crystalline environment. III. Oxides containing ionic complexes. *Phys. Rev. B* **2001**, *64*, 125117, doi:10.1103/PhysRevB.64.125117.

S5. Dorenbos, P. 5d-level energies of Ce^{3+} and the crystalline environment.IV. Aluminates and "simple" oxides. *J. Lumin.* **2002**, *99*, 283–299.

S6. Morrison, C.A. Host dependence of the rare-earth ion energy separation $4f^{N}$-$4f^{N-1}$ *nl*. *J. Chem. Phys.* **1980**, *72*, 1001–1002.

S7. Aull, B.F.; Jenssen, H.P. Impact of ion-host interactions on the 5d-to-4f spectra of lanthanide rare-earth-metal ions. I. A phenomenological crystal-field model. *Phys. Rev. B* **1986**, *34*, 6640–6646.

S8. Cotton, F.A. *Chemical Applications of Group Theory*, 3rd ed.; John Wiley & Sons, Inc.: New York, NY, USA, 1990; pp. 270–273.

S9. Lever, A.B.P. *Inorganic Electronic Spectroscopy*, 2nd ed.; Elseiver: Amsterdam, The Netherlands, 1984.

S10. Jarý, V.; Havlák, L.; Bárta, J.; Mihóková, E.; Nikl, M. Optical properties of Eu^{2+}-doped $KLuS_2$ phosphor. *Chem. Phys. Lett.* **2013**, *574*, 61–65.

S11. Stoll, S.; Schweiger, A. EasySpin, a comprehensive software package for spectral simulation and analysis in EPR. *J. Magn. Reson.* **2006**, *178*, 42–55.

S12. Laguta, V.; Buryi, M.; Havlák, L; Bárta, J.; Jarý, V.; Nikl, M. Stabilization of Eu^{2+} in $KLuS_2$ crystalline host: An EPR and optical study. *Phys. Status Solidi RRL* **2014**, *8*, 800–804.
